# Modulatory effect of metformin and its transporters on immune infiltration in tumor microenvironment: a bioinformatic study with experimental validation

**DOI:** 10.1007/s12672-025-02766-y

**Published:** 2025-05-31

**Authors:** Ahmed A. Rashad, Mohamed F. Elshafie, Safwat A. Mangoura, El-Sayed Akool

**Affiliations:** 1 Department of Clinical Pharmacy, Faculty of Pharmacy, Al-Azhar University, Nasr City, 4434104 Cairo Governorate Egypt; 2 Department of Clinical Pharmacy, Faculty of Pharmacy, Badr University in Cairo (BUC), Badr, Cairo 11829 Egypt; 3 Department of Pharmacology and Toxicology, Faculty of Pharmacy, Al-Azhar University, Cairo, Egypt

**Keywords:** Metformin, Organic cationic transporter, Tumor microenvironment, TIMER2.0, Immune infiltrates, Urokinase plasminogen activator

## Abstract

Metformin is a traditional antidiabetic drug for type 2 diabetes mellitus. However, it showed antitumor activity in many types of tumors, and it also has an influence on tumor metastasis in several types of tumors. It is transported through organic cationic transporters (OCTs), OCT1, OCT2, and OCT3, into the cells or into tumor microenvironment (TME). The complex interaction of metformin and its transporters on immune infiltration in TME of different types of tumors of The Cancer Genomic Atlas (TCGA) is not yet studied. The objective of this study is to identify the most suitable therapeutic target of tumors and immune infiltrates for metformin and its transporters in the TME. TIMER2.0, a bioinformatic tool, and other computational analysis were used to investigate this complex interaction; moreover, the identification of metformin target protein in TME is also investigated. The results revealed that the most suitable therapeutic target for metformin and OCTs among 32 types of TCGA data tumor types is Breast Invasive carcinoma (BRCA), and the most relevant immune infiltrate among 14 types of immune infiltrates that yields better prognosis and better therapeutical effect in TME is Macrophage M1. Furthermore, metformin showed a cytotoxic effect and an inhibitory effect on Urokinase Plasminogen Activator (uPA) gene expression in a concentration dependent fashion in MDA-MB-231 breast cancer cell line. This may suggest that metformin is a promising antitumor drug, stimulant for natural antitumor immune infiltrates, and inhibitor for metastasis in breast cancer.

**Supplementary Information:**

The online version contains supplementary material available at 10.1007/s12672-025-02766-y.

## Introduction

Metformin, a synthetic guanidine derivative first isolated from Galega officinalis in 1922, was approved as a medication in France (1957), England (1957), Canada (1972), and the U.S. (1995) [1, 2]. With over 60 years of clinical use, it remains a safe, affordable, and essential treatment for type 2 diabetes [1, 3]. Recent studies highlight its therapeutic benefits in obesity, metabolic syndrome, cancer, aging, neurological disorders, cardiovascular diseases, and polycystic ovarian syndrome [4]. In animal models, metformin alone or combined with radiation therapy inhibits tumor growth in melanoma, ovarian, prostate, and breast cancers [5–12]. Its mechanism involves AMPK activation (via ATM and LKB1), which suppresses mTOR activity, thereby inhibiting protein synthesis and cell proliferation [13].

The Tumor Microenvironment (TME) comprises a dynamic network of extracellular matrix components and diverse cells, including immune cells (macrophages, T cells, dendritic cells, B cells, natural killer cells), stromal cells (fibroblasts, pericytes, adipocytes), and vasculature [14]. Cancer, as a systemic disease, drives functional and compositional immune system alterations [15], with tissue immunity regulated by cross-talk among multiple cell lineages [16]. Understanding tumor immunology thus requires analyzing the systemic immune landscape within the TME. Advancing this knowledge demands technical innovations to decode immune cell interactions and phenotypes, leveraging data from resources like The Cancer Genome Atlas (TCGA) [17]. Metformin’s anticancer potential, initially observed in epidemiological studies linking it to reduced cancer risk [18], aligns with evidence that anticancer therapies often target both malignant cells and TME components, given the interdependent nature of tumors and their microenvironment [19].

Drug handling is carried out by a multitude of distinct transport proteins because each cell has a membrane that isolates it from its surroundings. Accordingly, transporters have an impact on human physiology and pathophysiology and are essential components for the therapeutic effect of a treatment [20]. Significantly, metformin absorption, distribution, and renal excretion are significantly influenced by membrane transporters. The pharmacokinetics of metformin are determined by a number of organic cationic transporters (OCT1, OCT2, and OCT3), several of which are crucial to the drug's pharmacological effect as mediators of metformin entrance into target tissues [21].

Metformin can generally lower the incidence, recurrence, and mortality of cancer, increase the responsiveness of cancer cells to treatment when incorporating chemotherapy and radiation, and optimize the migration and malignancy of tumors [18]. The theory of"seed and soil"describes how TME (the soil) and cancer cells (the seeds) interact and demonstrates how the"seeds"adjust to their"soil."[22]. Because of its antioxidant properties, metformin suppresses the immune system when paired with other medical conditions including inflammatory or autoimmune diseases [23]. Metformin appears to have immune-suppressive effects in various disorders as a result of its impact on immune cells [24]. On the other hand, other researchers indicated that metformin has indirect immune-stimulatory effects, and these immune-stimulating capabilities contribute to its anticancer efficacy [24, 25]. This is an indication for that metformin has an impact on homeostasis of the function of immune cells [26]. However, few is known about how metformin might affect different immune cells within the TME of different types of cancer. The complex interaction between metformin and its transporters in TME on immune cell infiltration is not yet investigated.

So, it’s hypothesized that the overexpression of transporters (OCT1 and/or OCT2 and/or OCT3) in the TME are accompanied with higher concentration of metformin. This may be a key predictor of metformin transit into TME as well as its immune cell infiltrate targets within TME to elicit natural immune response against tumor cells. Examining metformin's target proteins in TME and proteins particularly those responsible for tumor metastasis is crucial, and it may help us understand metformin’s antitumor effects. Consequently, this may shed light on the role of metformin in the activation of the natural immune response against tumor in different cancer types. Moreover, using bioinformatic tools will help identify the most promising tumor type and the most infiltrating immune cell in the TME as well as metformin target protein that affects tumor metastasis.

## Materials and methods

### Study design

To better understand how metformin does its antitumor effects and study the way it affects immune cells in TME, bioinformatic tool, TIMER2.0, is used to collect data about immune cells, genes, correlations between them, mutation rates of genes, and clinical impact of immune infiltrates and/or genes on tumor types. In this study, Immune Association and Cancer Exploration Components of TIMER2.0 were used. In Immune Association Component, Gene Module, Mutation Module, and Outcome Module were used to collect information about correlation between immune infiltrates and OCT genes in different 32 types of tumor, mutation rates of OCT genes, and the clinical outcomes of immune infiltrates in different tumor types, respectively.

An intersection between Gene Module and Outcome Module of Immune Association Component is made by using another bioinformatic tool (Venny) [27] to determine the most promising type of tumor that has positive correlation with OCT genes and the most infiltrating immune cell that possess a decrease risk effect, good clinical impact, on this tumor type.

In Cancer Exploration Component, Gene_DE Module and Gene_Outcome Module were used to detect if the expression of selected gene has different rate of expression in tumor tissue in comparison to normal adjacent tissue and to detect if selected gene expression independently has a good clinical impact on tumor types, respectively.

To determine if there is a specific human target protein in TME which is responsible for metastasis of tumor, another bioinformatic tool, SwissTargetPrediction, is used to detect the possible target proteins that can interact with metformin. To revalidate the result of SwissTargetPrediction, another bioinformatic tool, Way2 drug, is used to confirm the presence of possible interaction between metformin and the suggested target protein.

After determining the predicted target protein, Molecular Docking and Molecular Dynamic Simulation are done to evaluate the direct interaction between metformin and the target protein in TME and how stable is this interaction.

**Fig. 1:**
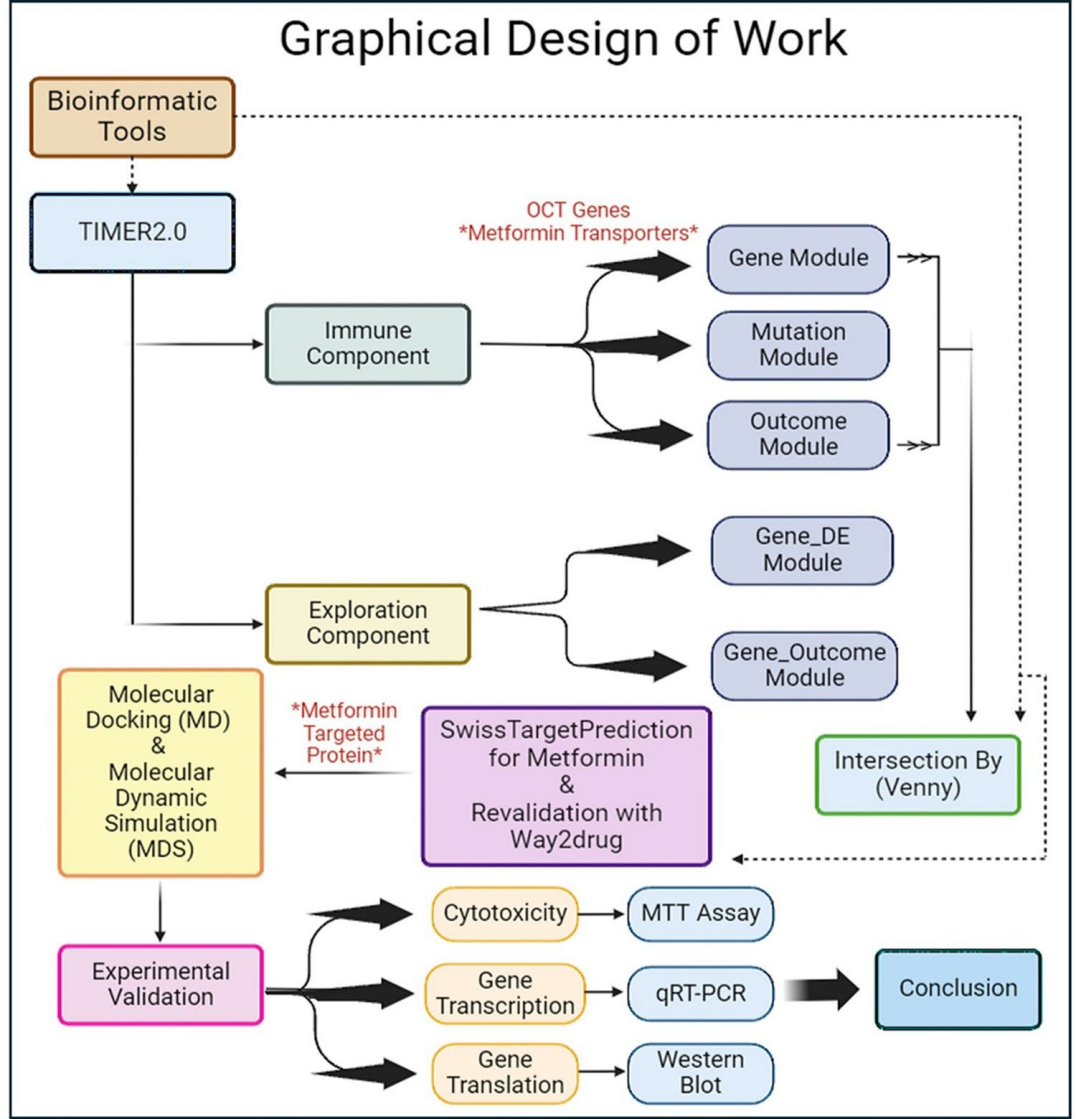
Graphical Design for the bioinformatic study of Metformin and experimental validation

To draw a conclusion to the study, experimental laboratory tests are undergone to analyze the effect of metformin on the target protein’s gene expression and cell viability. The graphical design of the work of this study is demonstrated by Fig. 1.

### Bioinformatic analysis

*The Cancer Genomic Atlas (TCGA)* framework is systematic, featuring numerous collaborating centers in charge of sample processing and collecting, high-throughput sequencing, and advanced bioinformatics data analysis [28]. TCGA, introduced in 2013, is one of the key initiatives employing advanced genome analysis technology to expedite our understanding of the genetics of cancer and aid in the development of novel cancer medicines, diagnostic techniques, and preventative measures [28–30].

*TIMER2.0, Tumor Immune Estimation Resource* (http://timer.cistrome.org/) is an internet server that is openly accessible to the scientific and research community and is powered by the Shiny web framework, which is R package that makes it easy to build interactive web applications [31, 32]. TIMER2.0 utilizes the data from TCGA to better study estimation of immune infiltrates levels, genetic features, and clinical features of these immune infiltrates [32]. The three principal components of TIMER2.0 are Immune Association, Cancer Exploration, and Immune Estimation [32].

Several modules will be used by TIMER2.0 through utilizing Clinical data among 11,000 samples and across 33 types of tumor from The Cancer Genomic Atlas (TCGA) database [30]. Although TCGA database includes 33 types of tumor, TIMER2.0 includes only 32 types of tumor along with 8 subtypes of these tumors and 10,897 tumor samples [33].

The 32 tumor types, and their 8 subtypes, from TIMER2.0 that are used in this study are Adrenocortical carcinoma (ACC), Bladder urothelial carcinoma (BLCA), Breast invasive carcinoma (BRCA), Breast invasive carcinoma—Basal (BRCA-Basal), Breast invasive carcinoma—Her2 (BRCA-Her2), Breast invasive carcinoma—LumA (BRCA-LumA), Breast invasive carcinoma—LumB (BRCA-LumB), Cervical and Endocervical Cancer (CESC), Cholangiocarcinoma (CHOL), Colon Adenocarcinoma (COAD), Diffuse Large B-cell Lymphoma (DLBC), Esophageal Carcinoma (ESCA), Glioblastoma Multiforme (GBM), Head and Neck Cancer (HNSC), Head and Neck Cancer—HPV- (HNSC-HPV-), Head and Neck Cancer—HPV + (HNSC-HPV +), Kidney Chromophobe (KICH), Kidney Renal Clear Cell Carcinoma (KIRC), Kidney Renal Papillary Cell Carcinoma (KIRP), Lower Grade Glioma (LGG), Liver Hepatocellular Carcinoma (LIHC), Lung Adenocarcinoma (LUAD), Lung Squamous Cell Carcinoma (LUSC), Mesothelioma (MESO), Ovarian Serous Cystadenocarcinoma (OV), Pancreatic Adenocarcinoma (PAAD), Pheochromocytoma and Paraganglioma (PCPG), Prostate Adenocarcinoma (PRAD), Rectum Adenocarcinoma (READ), Sarcoma (SARC), Skin Cutaneous Melanoma (SKCM), Skin Cutaneous Melanoma—Metastasis (SKCM-Metastasis), Skin Cutaneous Melanoma—Primary (SKCM-Primary), Stomach Adenocarcinoma (STAD), Testicular Germ Cell Tumors (TGCT), Thyroid Carcinoma (THCA), Thymoma (THYM), Uterine Corpus Endometrial Carcinoma (UCEC), Uterine Carsinosarcoma (UCS), and Uveal Melanoma (UVM).

Furthermore, the 14 immune infiltrates of interest used in this study are B cell, CD4+ T cell, CD8+ T cell, Dendritic cell, Macrophage M0, Macrophage M1, Macrophage M2, Mast cell, Monocyte, Natural Killer cell, Neutrophils, T cell Follicular Helper, T cell Gamma Delta, and T regulatory cell. These 14 types of immune infiltrates were selected to cover, as much as possible, the main components of the immune system functions. They are expressed as cell mediated immunity (CD4+ T cell, CD8+ T cell, T cell Follicular Helper, T cell Gamma Delta, and T regulatory cell), humoral immunity (B cell), phagocytic and antigen presenting cells (Macrophage M0, Macrophage M1, Macrophage M2, Monocyte, Neutrophils, Dendritic cell, and Mast cell), and anti-tumor activity (Natural Killer cell).

#### Immune association component in TIMER2.0

Among the modules of Immune Association component, this study used three types of modules: Gene Module, Mutation Module, and Outcome Module. CIBERSORT algorithm has been used due to its specificity in estimation of the intratumoral abundance of particular immune cell subsets and/or immune cell activation states [34].

##### Gene module

This module intends to visualize the correlation between gene expression and immune infiltration level in diverse cancer types. TIMER2.0 provides a heatmap table of the partial Spearman’s correlation to perform this association analysis [32]. In the TIMER2.0 website, there is an option to select the “Purity Adjustment” to be either involved in this correlation or not, referring to this Tumor Purity option determines the proportion of tumor cells in the TME. In our study, we used the Gene Module to test the relation between our genes of interest (OCT1, OCT2, and OCT3) and the 14 different types of immune infiltrates of interest while activating the “Purity Adjustment” option [35, 36].

##### Outcome module

Outcome Module in TIMER2.0 could rapidly assess the relationship between immune cell prevalence and patient survival, sometimes mentioned as the clinical impact or clinical relevance, for all TCGA cancer types [32].

##### Bioinformatic intersection analyses between gene expression and infiltration of immune cells within TME as well as clinical impact of infiltration of immune cells on patient survival

The purpose of this analysis is to assign the most prevalent types of tumor that show high expression of OCT genes through which the immune infiltrates can pass through, by the help of metformin, into the TME to eradicate the tumor and the most effective immune infiltrates that show decreased risk, or better clinical outcome, in most of the types of TCGA 32 types of tumor. To perform these intersections, we used a bioinformatic tool"Venny"(https://bioinfogp.cnb.csic.es/tools/venny/) [27] to detect the similarities and the number of occurrences of both immune infiltrates in each type of cancer and types of cancer in which the immune infiltrates were introduced.

##### Mutation module

When the gene of interest is selected, TIMER2.0 shows a bar plot illustrating the frequency of gene mutations in TCGA tumor types [32].

#### Exploration component

TIMER2.0 features the Cancer Exploration Component with four modules to study cancer-related associations among tumor features in the TCGA data [32]. In this component, we used only 2 modules, which are Gene_DE and Gene_Outcome.

##### Gene_DE module

This module in TIMER2.0 enables us to easily compare the Differential Expression (DE) level of a gene between normal tissues and tumors for TCGA tumor types, where the clinical significance of the results are determined by differential gene expression using edgeR software [32].

##### Gene_Outcome module

TIMER2.0 includes Gene_Outcome, that assess the relationship between several gene’s expression and patient survival for tumor types of TCGA data [32, 37]. TIMER2.0 creates a heatmap table of the normalized coefficients of the input gene expression in the Cox model as soon as the user makes the request. We used our genes of interest (OCT1, OCT2, OCT3 genes) to detect the patient’s survival among TCGA tumor types.

#### Prediction of metformin target

To help us predict the possible protein targets that affect tumor metastasis, SwissTargetPrediction (http://www.swisstargetprediction.ch) [38] is a bioinformatic and online web tool that try to predict and discover a target using ligand-based approach for any bioactive small molecule. We subjected metformin into this bioinformatic tool to find the possible target proteins that can interact with metformin and search if any of the predicted targets might be effective in the TME. For further validation, another prediction bioinformatic tool (Way2 drug) [39] is used to confirm the results obtained from SwissTargetPrediction.

### Docking and molecular dynamic simulation (MDS)

The structure of human Urokinase Plasminogen Activator (uPA) in complex with urokinase receptor (PDB code: 2 FD6) was downloaded from the protein databank (RCSB) and the uPA protein chain A was extracted from the structure and used for the molecular docking and molecular dynamic simulation (MDS).

#### Protein preparation

The protein was prepared using the Protein Preparation Workflow impeded with Maestro version 13.7.125, MMshare Version 6.3.125, Release 2023–3, Platform Linux-x86_64, Schrodinger suite (version2023-3). These all are computer programs that work all together at the same time to elaborate the possible molecular interaction of metformin with uPA protein. The docking grid box was built around the domain interaction with uPA (chain A in the PDB code: 2 FD6) using the grid generation method (Schrodinger suite version2023-3).

#### Metformin docking

Metformin chemical structure was built using the 2D sketcher in Maestro and the LigPrep wizard (Schrodinger suite version2023-3) was used to prepare it for docking. Metformin was docked onto the identified site using the XP (extra precision) mode and performing post docking minimization.

#### Metformin poses MDS

The top ranked poses were chosen to be evaluated by MDS. The rest of the predicted poses that showed unfavorable scores (positive values) were discarded and not considered for further analysis.

MDS was performed using the Desmond module of the Schrodinger suite (version2023-3). All systems were solvated in an orthorhombic box (a margin of 10 Å between the solute and the side of the box was used in each dimension) with explicit TIP3P water molecules. All systems were neutralized, and anionic salt concentration of 0.15 M of NaCl was added. Atomistic interactions were calculated with the OPLS4 forcefield (Schrodinger 2023–3). Before equilibration and the long production MDS, the systems were minimized and pre-equilibrated using the default relaxation routine implemented in Desmond. A multiple time-stepping of 2, 2, and 6 fs was used. The system equilibration was done via NVT and NPT ensembles using the SHAKE algorithm and by bringing the temperature to 300 K and pressure to 1 bar. Then, the systems were submitted in 10 and 50 ns MDS for equilibration and production MD runs for each system. Finally, a 250 ns unconstrained MDS was performed for the system, and the coordinates were saved for every 5 ps.

### Experimental analysis

#### Metformin cytotoxicity in MDA-MB-231 human breast cancer cell line

Metformin was purchased from Utopia Pharmaceuticals (Cairo, Egypt) in a form of HCl salt. The cytotoxic activity of metformin was evaluated on MDA-MB-231 breast cancer cell line using MTT Assay Kit from Abcam (code: ab211091). The MDA-MB-231 cell line used in this study was obtained from American Type Culture Collection (ATCC, Manassas, USA, https://www.atcc.org/products/htb-26), and it was incubated in 5% carbon dioxide incubator at 37 °C. According to Horiuchi et al. [40], MDA-MB-231 cells (1 × 10^5^ cells/well) were plated in 0.2 ml of medium/well in 96-well plates, and treated with distinct doses of metformin (1000, 3000, 5000, 7000, and 9000μM) in triplicate cultures. After the incubation period 48 h, the medium was removed from the wells carefully. Then, each well has been washed by DMEM FCS medium for 2–3 times, and then 200µl of MTT (5 mg/ml) dye was added. Afterwards, the incubation plates were left in 5% CO2 incubator for 6–7 h to induce cytotoxicity. After that, Dimethyl Sulfoxide (DMSO), a solubilizing agent, was added to each well, mixed by micropipette, and left for 45 s. Directly proportional to the number of viable cells in the media, formazan violet crystals were visible and ready to be evaluated by colorimetric methods. The optical density (OD) values were measured at 595nm. Measurements were performed and collected, and the IC50 was determined graphically [40], using the IC50 Quest Graph™ Calculator in AAT Bioquest (https://www.aatbio.com/tools/ic50-calculator) [41].

#### Evaluation of the effect of metformin on uPA gene expression using qRT-PCR and its translation by Western Blot analysis

MDA-MB-231 cells were incubated in 6-well plates in triplicate cultures for 48 h following the addition of metformin in concentrations of 0%, 10%, and 20% of IC50 at 37 °C in CO2 incubator. After incubation, the cells underwent trypsinization, resuspended, and centrifuged at 1000 rpm for 10 min. The cell pellets were collected and subjected to qRT-PCR to determine the uPA gene transcription while the supernatants were subjected to by Western Blot analysis to evaluate the protein level of uPA.

##### Quantitative real-time polymerase chain reaction (qRT-PCR)

Total RNA was extracted using TRIzol™ reagent (Catalog number: 15596026, Invitrogen™—Life Technologies Inc, Thermo Fischer, USA). Then, RNA was reverse transcribed into complementary DNA (cDNA) using QuantiTec Reverse Transcriptase Kit (Catalog number: 205311, Qiagen, USA). The Maximas SYBR Green/Fluorescein qPCR Master Mix (Catalog number: K0241, Invitrogen™—Life Technologies Inc, Thermo Fischer, USA) was used to cDNA amplification. The amplification reaction mixture (25 µl) is composed of Maximas SYBR Green/Fluorescein qPCR Master Mix 12.5 μl, forward primer 0.3 μl, reverse primer 0.3 μl, and finally fill up to 25 μl with nuclease free water. Then, the cDNA is added in a less than or equal to 100 ng/reaction. Forty cycles of the following were performed on the samples in the RT-PCR apparatus: initial denaturation (95 °C, 3 min), denaturation (95 °C, 30 s), annealing (30 s), extension (72 °C, 30 s), and final extension (72 °C, 5 min).

According to Livak and Schmittgen [42], the comparative cycle threshold (Ct) (2^**−∆∆Ct**^) was used to analyze the resulting data and calculate the relative expression. Expression levels were normalized to Glyceraldehyde-3-phosphate dehydrogenase. The primer used for uPA gene is 5′-GACCCCCTCGTCTGTTCCCTCCAAG-3′ (forward) and 5′-CTCTTCCTTGGTGTGACTGCGG −3′ (reverse), and the primer of GAPDH is 5′-GTCGCTGTTGAAGTCAGAGGAG-3′ (forward) and 5′-CGTGTCAGTGGTGGACCTGAC-3′ (reverse) [43].

##### Western Blot

The uPA protein level in conditioned media was analyzed by Western Blot analysis. Briefly, protein extracts were denatured by Laemmli sample buffer. Afterwards, the protein extracts were separated using 10% sodium dodecyl sulphate (SDS)—polyacrylamide gel electrophoresis [44, 45].

##### Statistical analysis

To test the significance of the results obtained from experimental validation tests, the statistical analysis for qRT-PCR and Western Blot was done by One Way ANOVA and Tukey Kramer as Post ANOVA test. The Significance was accepted at *p* ≤ 0.05.

## Results

### Bioinformatic analysis

#### Immune association component

##### Gene module

Our genes of interest (OCT1, OCT2, and OCT3) showed positive, negative, or non-significant (*p*-value > 0.05) correlations with our immune infiltrates of interest based on the 32 TCGA tumor types selected in this module, and all the results are collected in the supporting files in Supplementary Information section, *Online Resource 1*. In case of OCT1, OCT2, OCT3 genes, the immune infiltrates that show the highest number of positive correlation results with these genes in different tumor types are T regulatory Cells, 12 times; Macrophage M1, 14 times; Bcell, 17 times; Mast Cell, 17 times; and Macrophage M0, 18 times, all found in Table 1A.

**Table 1:** Tumors showing highest positive correlation of OCT genes expressions with 14 immune infiltrates in 32 types of tumor of TCGA data

(A)		(B)	
Immune Infiltrates	Frequency of immune infiltrates presence in tumor types that show a positive correlation with OCT1, OCT2, and OCT3 genes expressions in 32 tumor types of TCGA data	Tumor Types	Frequency of tumor types presence with immune infiltrates that show a positive correlation with OCT1, OCT2, and OCT3 genes expressions in 32 tumor types of TCGA data
Macrophage M0	18	KIRC, LUAD	7
Bcell, Mast Cell	17	BRCA, THYM, SKCM, SKCM-Metastasis, LUSC, LIHC	6
Macrophage M1	14	BLCA, BRCA-LumA, COAD, OV, SARC	5
Treg Cell	12		

(A) Immune infiltrates with highest frequency of presence. (B) Tumor types with highest frequency of presence

The tumor types that show the highest number of positive correlations with OCT genes are BLCA, BRCA-LumA, COAD, OV, and SARC, 5 times each; BRCA, LIHC, LUSC, SKCM-Metastasis, SKCM, and THYM, 6 times each; and KIRC and LUAD, 7 times each, mentioned in Table 1B. All the data about the results of all immune infiltrates and tumor types with positive correlation with OCT genes are found in *Online Resource 2*. Therefore, the aim is to identify the immune infiltrates that are positively correlated to OCT genes, have decrease risk clinical outcome, and are found in the tumor types that show positive correlation with OCT genes.

Regarding tumor purity, BRCA, BRCA-LumA, OV, THYM, BLCA, and LUSC tumors showed negative tumor purity result in OCT1, OCT2, and OCT3 genes all together, Table 2.

**Table 2:** Tumor purity results in OCT1, OCT2, and OCT3 genes of tumor types that show highest frequency of positive correlation with OCT genes expressions in 32 tumor types of TCGA data

Tumor types with immune infiltrates that show highest frequency of positive correlation with OCT1, OCT2, and OCT3 genes expressions in 32 tumor types of TCGA data	OCT1	OCT2	OCT3	Total number of negative Rho result (Rho < 0)
	Tumor Purity (Rho Results)			
LUAD	− 0.09	− 0.052	0.006	2
KIRC	− 0.042	− 0.002	0.006	2
BRCA	− 0.076	− 0.198	− 0.0471	3
SKCM-Metastasis	0.013	− 0.191	− 0.515	2
SKCM	0.018	− 0.132	− 0.469	2
THYM	− 0.128	− 0.096	− 0.04	3
LUSC	− 0.041	− 0.083	− 0.35	3
LIHC	− 0.041	0.103	0.055	1
BLCA	− 0.104	− 0.335	− 0.447	3
BRCA-LumA	− 0.057	− 0.197	− 0.502	3
OV	− 0.049	− 0.216	− 0.387	3
COAD	− 0.059	− 0.143	0.107	2
SARC	− 0.009	0.214	0.304	1

Results of Rho are obtained from Gene Module in Immune Association Component of TIMER2.0, found in Supporting file 1. Rho < 0 means low tumor purity and thus low proportion of tumor cells and higher proportion of immune cells in TME, all leading to better prognosis

##### Outcome module

**Fig. 2:**
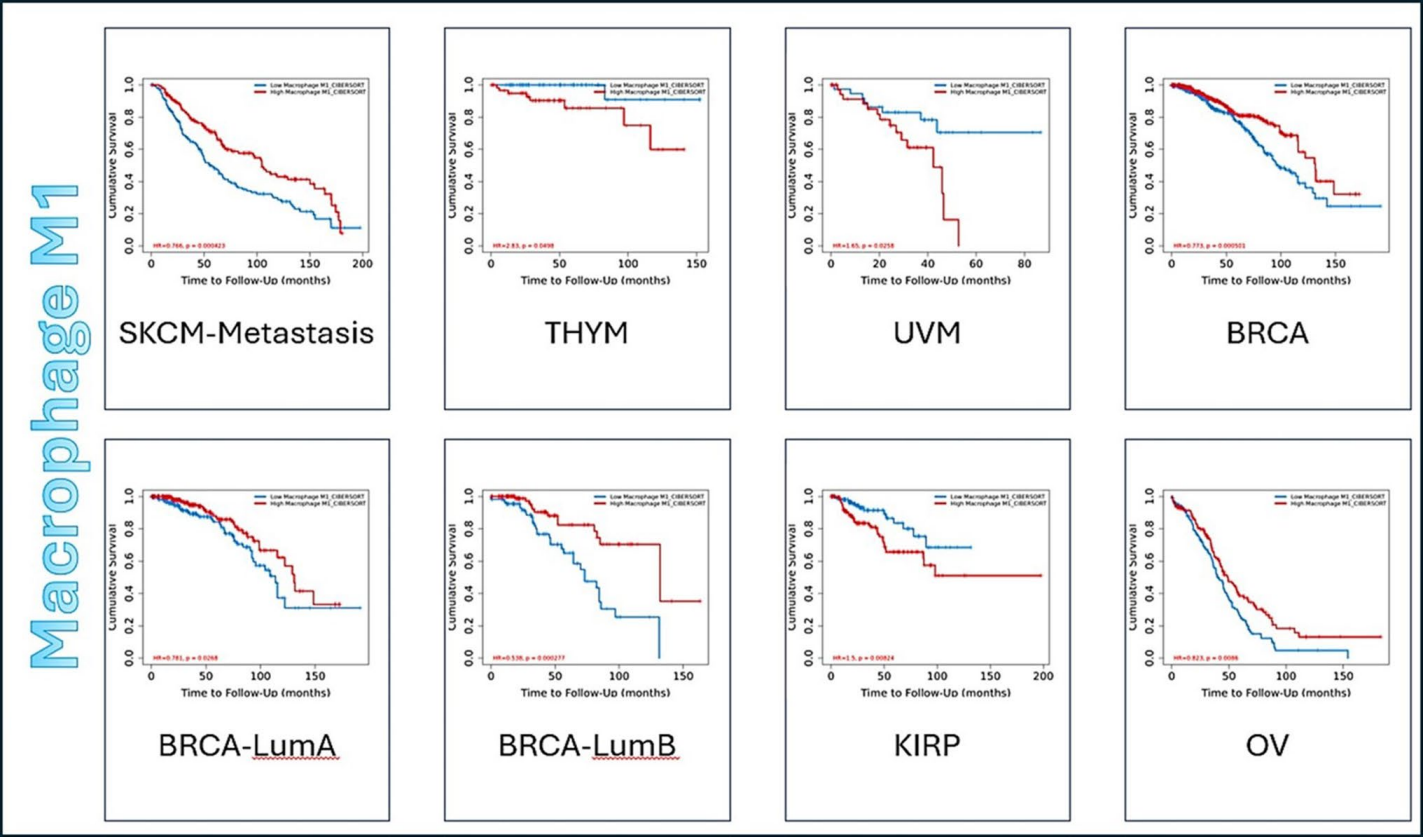
Representative figure for the Outcome module of Macrophage M1, as an example for the 14 immune infiltrates, against 32 tumor types of TCGA data. Kaplan–Meier Curve displays immune infiltrate clinical outcome on tumor types. Split Infiltration Percentage of Patients = 50%. Survival Time Between in months = 200. Significant result (*p*−value < 0.05). Increase risk result (Z score > 0) and decrease risk (Z score < 0)

The clinical impact of immune infiltrates is expressed in Kaplan–Meier Curve with parameters adjusted as following: Split Infiltration Percentage of Patients is 50% and Survival Time Between is 200 months. The longer the survival time of the data the higher is the data’s credibility. Z-score discriminates between the increase risk (Z > 0) and decreased risk (Z < 0) results. Figure 2 shows an example of 3 types of tumor displayed by Kaplan–Meier Curve for Macrophage M1 immune infiltrate as an example for the 14 immune infiltrates, while *Online Resource 3* has all the figures of all 14 immune infiltrates, showing only the significant results (*p*-value < 0.05).

The results of clinical outcomes of all 14 immune infiltrates are mentioned in *Online Resource 4*. The infiltrates that showed highest frequency of decrease risk results, regarded as safe infiltrates, are CD8 + Tcell (found in SKCM, HNSC, LIHC, BLCA, DLBC, BRCA, SKCM-Primary, SKCM-Metastasis, CESC, and HNSC HPV + ve), Macrophage M1 (found in SKCM, BRCA, BRCA-LumA, BRCA-LumB, OV, and SKCM-Metastasis), and Tcell Follicular Helper (found in STAD, THCA, OV, BRCA, and BRCA-LumA). On the other hand, the immune infiltrate that showed highest frequency of increase risk (marked as pro-tumor because it can promote tumor progression) is Macrophage M2 [46]. Results are displayed in Table 3 where it summarizes the frequency of significant results of immune infiltrates that show either increase risk or decrease risk in all 32 tumor types of TCGA data.

**Table 3:** The clinical outcome results of 14 immune infiltrates in 32 types of tumor in TCGA data

	The immune infiltrate	Significant result frequency	
		Increase risk	Decrease risk
1	Mast cell	0	3
2	Dendritic cell	2	1
3	B cell	1	3
4	Neutrophil	3	1
5	Monocyte	1	2
6	Macrophage M0	4	2
7	Macrophage M1	3	6
8	Macrophage M2	8	1
9	Natural killer cell	3	2
10	Treg cell	1	4
11	CD4 + Tcell	2	2
12	CD8 + Tcell	3	10
13	Tcell Gamma Delta	1	3
14	Tcell Follicular Helper	1	5

Analysis was done using TIMER2.0 without gene specification or clinical covariates

Significant means *p* value < 0.05. Increase Risk (red color) means Z-score > 0. Decrease Risk (blue color) means Z-score < 0

##### Bioinformatic intersection analyses between gene expression and infiltration of immune cells within TME as well as Clinical impact of infiltration of immune cells on patient survival

As demonstrated in Table 4, Macrophage M1, which possesses decrease risk effect, has the highest presence in 4 different tumor types of TCGA types of tumor as decrease risk immune infiltrate, and that BRCA has the highest presence of immune infiltrates, among them is Macrophage M1, that show good clinical outcome in the TME, conferring that BRCA is the most promising tumor type that shows the best clinical outcome when OCT genes are provoked to let immune infiltrates, specifically Macrophage M1, enter into the TME.

**Table 4:** The frequency of intersection between both increase and decrease risk immune infiltrates and types of tumor that show positive correlation with OCT genes

(A)			(B)			
Tumor Type	Number of Occurrences of immune infiltrates in each tumor type	Types of Immune Infiltrates in each tumor type	Immune Infiltrate	Number of Occurrences of immune infiltrate in all tumor types		Types of tumor that immune infiltrates are found in
BRCA	4	Macrophage M1, Treg Cells, Tcell Gamma Delta, Tcell Follicular Helper	Macrophage M1	**↑1**	*↓4*	*BRCA, BRCA-LumA, SKCM*, **THYM**, *SKCM-Metastasis*
				Total = 5		
BRCA-LumA	3	Macrophage M1, Macrophage M0, CD8 + T Cell	NK Cells	**↑2**	*↓1*	*SARC*, **UVM, KIRC**
				Total = 3		
SKCM	3	Macrophage M1, CD4 + Tcell, CD8 + T Cell	CD4 + Tcell	**↑1**	*↓2*	**SKCM**, *THYM, LGG*
				Total = 3		
THYM	3	Macrophage M1, Macrophage M2, CD4 + Tcell	Monocyte	**↑1**	*↓1*	*UVM*, **LUSC**
				Total = 2		
SARC	2	Mast Cell, NK Cells	Macrophage M0	**↑1**	*↓1*	*BRCA-LumA*, **ACC**
				Total = 2		
UVM	2	Monocyte, NK Cells	CD8 + T Cell	**↑0**	*↓2*	*BRCA-LumA, SKCM*
				Total = 2		
LIHC	1	Neutrophil	Tcell Follicular Helper	**↑0**	*↓2*	*BRCA, THCA*
				Total = 2		
LUSC	1	Monocyte	Mast Cell	**↑0**	*↓1*	*SARC*
				Total = 1		
ACC	1	Macrophage M0	Neutrophil	**↑1**	*↓0*	**LIHC**
				Total = 1		
SKCM-Metastasis	1	Macrophage M1	Macrophage M2	**↑1**	*↓0*	**THYM**
				Total = 1		
KIRC	1	NK Cells	Treg Cells	**↑0**	*↓1*	*BRCA*
				Total = 1		
LGG	1	CD4 + Tcell	Tcell Gamma Delta	**↑0**	*↓1*	*BRCA*
				Total = 1		
THCA	1	Tcell Follicular Helper	Dendritic Cell	0		
			B Cell	0		

Intersection was done by Venny Website (https://bioinfogp.cnb.csic.es/tools/venny/). **↑** means increase risk. ↓ means decrease risk. Bold colored tumor indicates that the adjacent immune infiltrate has an increase risk effect in this tumor. Italic colored tumor indicates that the adjacent immune infiltrate has a decrease risk effect in this tumor. A) The frequency of presence of immune infiltrates in each type of tumor. B) The presence frequency and clinical outcome, both increase or decrease risk, of each immune infiltrate in tumor types

On the other hand, the second intersection is done between the same types of tumor that show positive correlation with OCT genes and all of the immune infiltrates that show significant clinical impact either increase or decrease risk, shown in Table 4.

As shown in Table 4, Macrophage M1, again, expressed the highest prevalence, total 5 times, in all tumor types with 4 times as decrease risk and once as increase risk and found that BRCA also showed the highest frequency of immune infiltration with the same immune infiltrates as those in decrease risk only. All results of intersections between immune infiltrates and positive correlated types of tumors are done by Venny and displayed in *Online Resource 5*, while the tables that analyze the obtained results and the raw data are found in *Online Resource 6*. The results obtained from Venny represent the intersection between Immune infiltrates that show decrease risk along with 32 types of tumors that show positive correlation with OCT genes in (SI. 1), while the correlation of Immune infiltrates that show the total of both increase and decrease risk clinical outcome along with the same 32 types of tumors that show positive correlation with OCT genes in (SI. 2) in *Online Resource 5*. These results reveal that Macrophage M1 is the immune infiltrate that showed the best clinical outcome in BRCA.

##### Mutation module

**Fig. 3:**
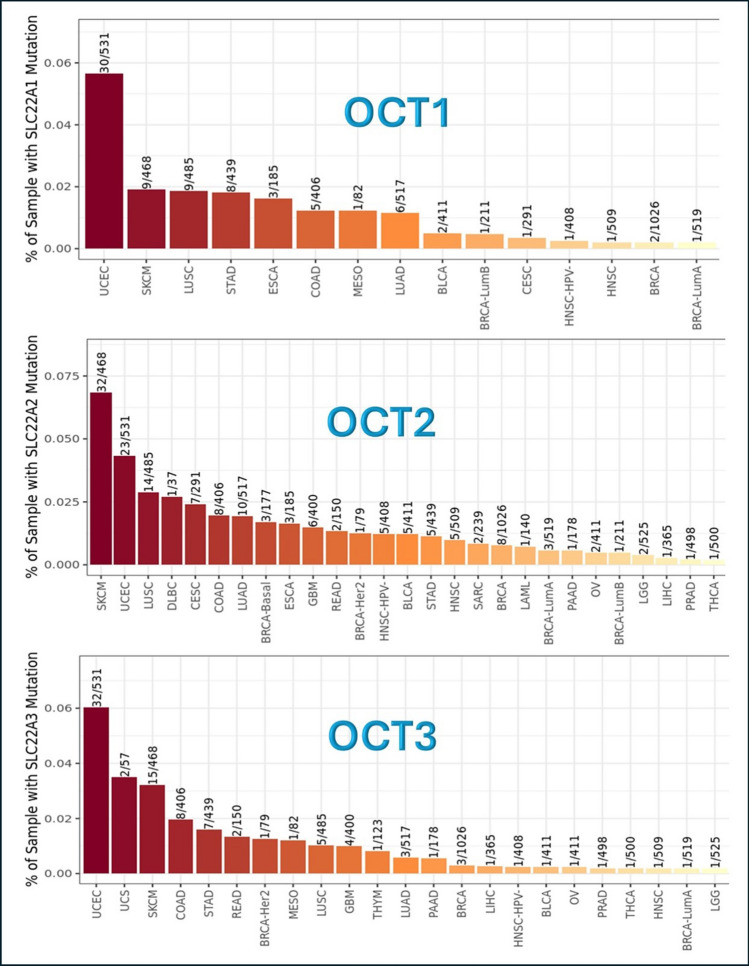
Frequency of OCT genes mutations in different tumor types. This analysis was done using Mutation module in TIMER2.0 website (http://timer.cistrome.org/). SLC22 A1 or OCT1 = Organic Cationic Transporter 1. SLC22 A2 or OCT2 = Organic Cationic Transporter 2. SLC22 A3 or OCT3 = Organic Cationic Transporter 3

When OCT genes are inserted into this module in TIMER2.0, a bar plot is executed referring to the frequency of mutation of the selected genes in different tumor types. The results are displayed in Fig. 3, where OCT1, OCT2, OCT3 genes, and their mutation frequency are expressed by ratio of presence.

The tumors that show the highest frequency of OCT1 gene mutation are UCEC and SKCM, while the lowest frequency tumors are BRCA, BRCA-LumA, and HNSC. The tumors that show the highest frequency of OCT2 gene mutation are UCEC and SKCM, while the lowest frequency tumors are BRCA, BRCA-LumA, BRCA-LumB, LAML, PAAD, OV, LGG, LIHC, PRAD, and THCA. Concerning OCT3 gene, the tumors that show the highest frequency of OCT3 gene mutation are UCEC, SKCM, and UCS, while the lowest frequency are BRCA, BRCA-LumA, LIHC, BLCA, HNSC-HPV-, OV, PAAD, THCA, HNSC, and LGG, Fig. 3.

All the results show that BRCA is among the lowest mutation frequency of tumor types in OCT1, OCT2, and OCT3 genes.

#### Exploration module

##### Gene_DE module

**Fig. 4:**
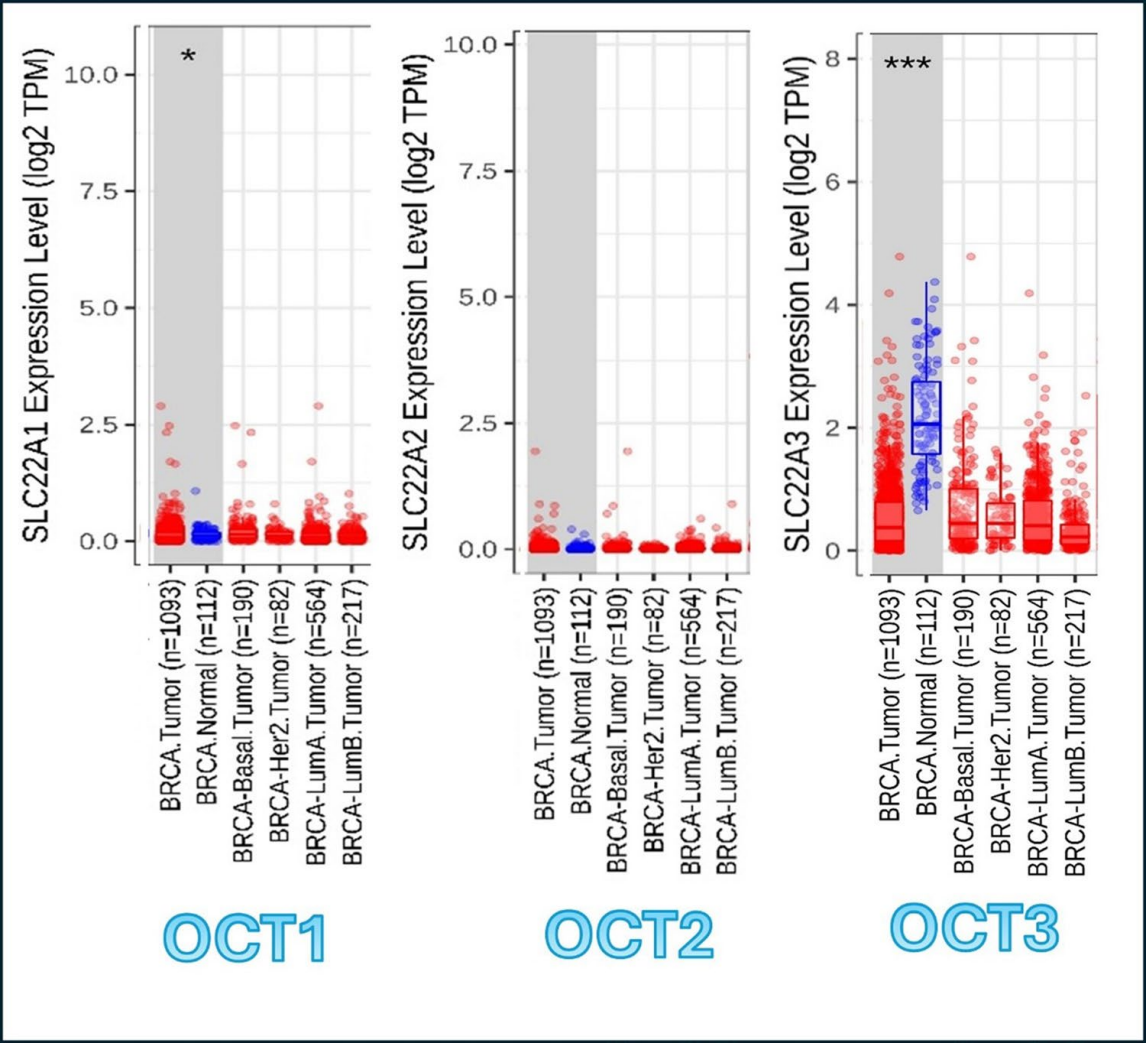
OCT genes expression in BRCA as compared to normal controls. The analysis was done by using Gene_DE module in TIMER2.0. Statistical significance computed by the Wilcoxon test that is annotated by the number of stars (*p−value < 0.05; ***p−value < 0.001). SLC22A1 or OCT1 = Organic Cationic Transporter 1. SLC22A2 or OCT2 = Organic Cationic Transporter 2. SLC22A3 or OCT3 = Organic Cationic Transporter 3. BRCA Normal = Breast normal tissue

In this module, genes that are up-regulated or down-regulated in the tumors can be identified and compared to normal tissues for each cancer type among the tumor types, as displayed in gray columns in *Online Resource 7* that shows the expression of OCT1, OCT2, and OCT3, respectively. In Fig. 4, the results of OCT1, OCT2, and OCT3 genes are displayed along with breast tumors (BRCA) only. The result shows the statistical significance computed by the Wilcoxon test that is annotated by the number of stars (*p-value < 0.05; ***p-value < 0.001). This statistical significance is determined by differential gene expression analysis using edgeR software.

Regarding to BRCA, the expression of OCT1 shows partial significant (partial higher) expression in BRCA tissue in comparison to the normal breast tissues. OCT2 shows no significant difference between normal and tumor tissues in expression of OCT2 gene; however, OCT3 shows the most significant result of expression of OCT3 in BRCA tissues against normal tissue in breast.

This means that OCT1 and OCT3 are more expressed in BRCA tissue as compared to the normal breast tissue, giving a better chance to uptake metformin and Macrophage M1 in breast TME.

##### Gene_Outcome module

This module provides the clinical relevance of OCT genes expressions along different tumor types in TCGA data. TIMER2.0 provides the opportunity to correlate between gene expression and one or more of clinical factors (e.g. age, tumor stage, gender, purity, and race), but it was not designed to compare between them to avoid specificity of any of these clinical factors. The whole results of this module are found in *Online Resource 8*, while all the significant results only (*p*-value < 0.05) are shown in Table 5 where the split expression percentage of patients is equal to 50%, and survival time between is 200 months to enable a better reliability of results of the module. The risk is identified by the Z score of the Cox proportional hazard model whether increase risk (red colored, Z > 0) or decrease risk (blue colored, Z < 0).

**Table 5:** Clinical outcome of OCT genes expressions in 32 tumor types of TCGA data

Tumor Types	OCT-1	OCT-2	OCT-3
	*p*-Values (< 0.05)		
ACC	**0.0412**	0.186	**0.000219**
BLCA	*0.0158*	0.108	**0.0113**
BRCA	0.243	*0.00946*	0.487
BRCA-LumA	**0.0231**	0.141	0.653
KIRC	0.0758	*1.47E-08*	*0.0147*
KIRP	0.441	*0.00805*	0.106
MESO	**0.0101**	0.355	**0.0262**
OV	0.254	0.0205	**0.0422**
SKCM	0.637	*0.00504*	0.0849
SKCM-Metastasis	0.567	*0.00481*	*0.0222*
THCA	0.131	0.368	**0.0422**

Data was expressed by Gene_Outcome Module in TIMER2.0 website (http://timer.cistrome.org/). Significant results (*p*-value < 0.05) of the clinical relevance of OCT1, OCT2, and OCT3 genes expression in different tumor types. Survival Time Between up to 200 + Months. Split expression percentage of patients = 50% by Kaplan Meier Curve. Increase risk (bold) means Z-score > 0. Decrease risk (italic) means Z-score < 0

Regarding the results collected, only OCT2 gene expression showed a significant decrease risk result in presence of BRCA, while OCT1 and OCT3 gene expressions showed non-significant results.

#### Prediction of metformin target

**Fig. 5:**
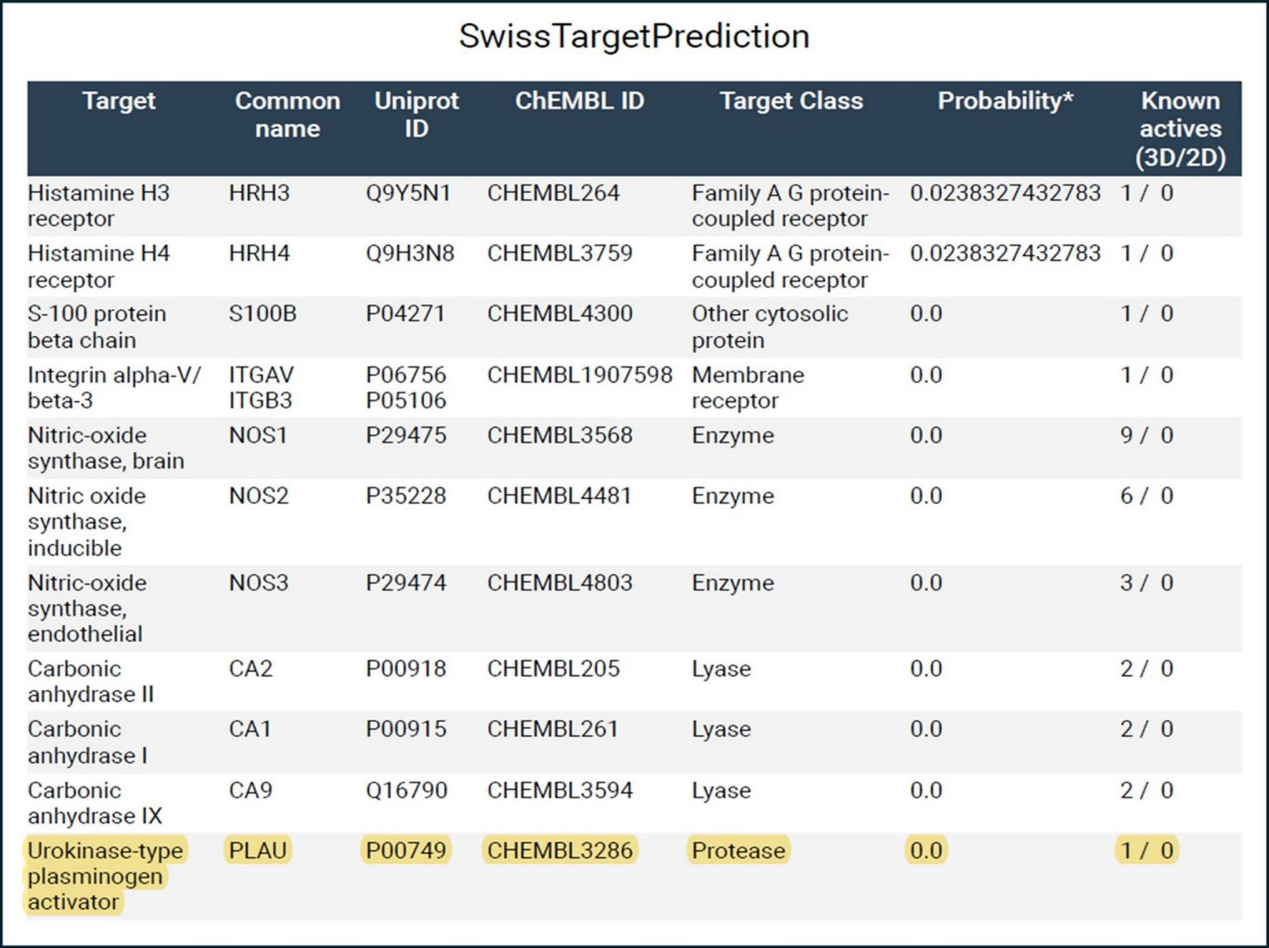
Possible Metformin target proteins. SwissTargetPrediction website has been used to elucidate the predicted results. Chembl ID (CHEMBL3286) is the id number of Urokinase−type plasminogen activator (uPA) in ChEMBL database website (https://www.ebi.ac.uk/chembl/target_report_card/CHEMBL3286/). Uniprot ID (P00749) is the id number of Urokinase−type plasminogen activator (uPA) in Uniprot website (https://www.uniprot.org/uniprotkb/P00749/entry.). Plasminogen Activator Urokinase (PLAU) = Urokinase−type Plasminogen Activator (uPA)

In SwissTargetPrediction website, Homo sapiens, refers to human, has been selected in which metformin would attach to any target protein. Possible interactions of metformin were shown in Fig. 5. The uPA gene, as one of the players of tumor metastasis, was chosen among the predicted targets to metformin.

Also, by another confirmatory bioinformatic tool, Way2 drug confirmed the presence of uPA gene (CHEMBL3286) as a possible interacting target with metformin by a confidence of 0.0445, and the results are shown in *Online Resource 9*.

#### uPA mutation module

**Fig. 6:**
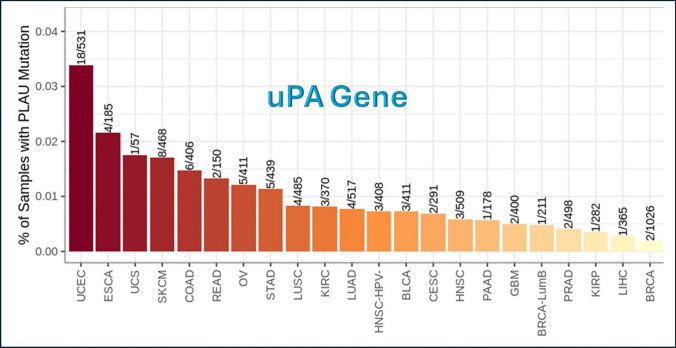
Frequency of uPA gene mutations in different tumor types. This analysis was done using Mutation module in TIMER2.0 website (http://timer.cistrome.org/). PLAU = uPA = Urokinase Plasminogen Activator

uPA gene underwent the Mutation module of Immune Association component, and Fig. 6 shows the frequency of mutation of uPA gene in different tumor types of TCGA data. The tumors that show the highest frequency of uPA gene mutation are UCEC and ESCA, while the lowest frequency are BRCA and LIHC.

This means that uPA gene is majorly wild type in BRCA, and that supports the sustainability of the obtained results.

### Docking and molecular dynamic simulation (MDS)

#### Metformin docking with uPA gene

According to the docking scores of metformin molecule with uPA protein, the three most negative poses are selected for further docking and MDS results Table 6.

**Table 6:** Docking scores for metformin with PLAU protein

	Title	Test 0	Flags	Ionization Penalty	State Penalty	XP GScore	Potential Energy—S—OPLS	Docking Score	Glide emodel	Glide gscore
	2 FD6-proteinprep_1 (1)									
1	2 FD6—5-removed waters						− 1296.916			
2	2 FD6—5-removed waters						− 1296.916			
**3**	**Metformin**	**1**	**0**	**0.0111**	**0.0015**	** − 4.134**		** − 4.132**	** − 17.867**	** − 4.134**
**4**	**Metformin**	**1**	**0**	**0.0111**	**0.0015**	** − 3.727**		** − 3.726**	** − 15.771**	** − 3.727**
**5**	**Metformin**	**1**	**0**	**0.0111**	**0.0015**	** − 2.437**		** − 2.436**	** − 17.074**	** − 2.437**
6	Metformin	1	0	0.0111	0.0015	− 2.426		− 2.424	− 17.787	− 2.426
7	Metformin	1	0	0.0111	0.0015	− 2.284		− 2.282	− 16.193	− 2.284
8	Metformin	1	0	0.0111	0.0015	− 2.174		− 2.173	− 17.354	− 2.174
9	Metformin	1	0	0.0111	0.0015	− 2.016		− 2.015	− 14.503	− 2.016
10	Metformin	1	0	0.0111	0.0015	− 1.877		− 1.875	− 19.186	− 1.877

Poses of interaction of metformin with PLAU protein were arranged from the most negative (strongest binding affinity) to the most positive (weakest binding affinity) using Schrodinger suite version2023-3. The three chosen poses are the first three poses highlighted with bold

**Fig. 7:**
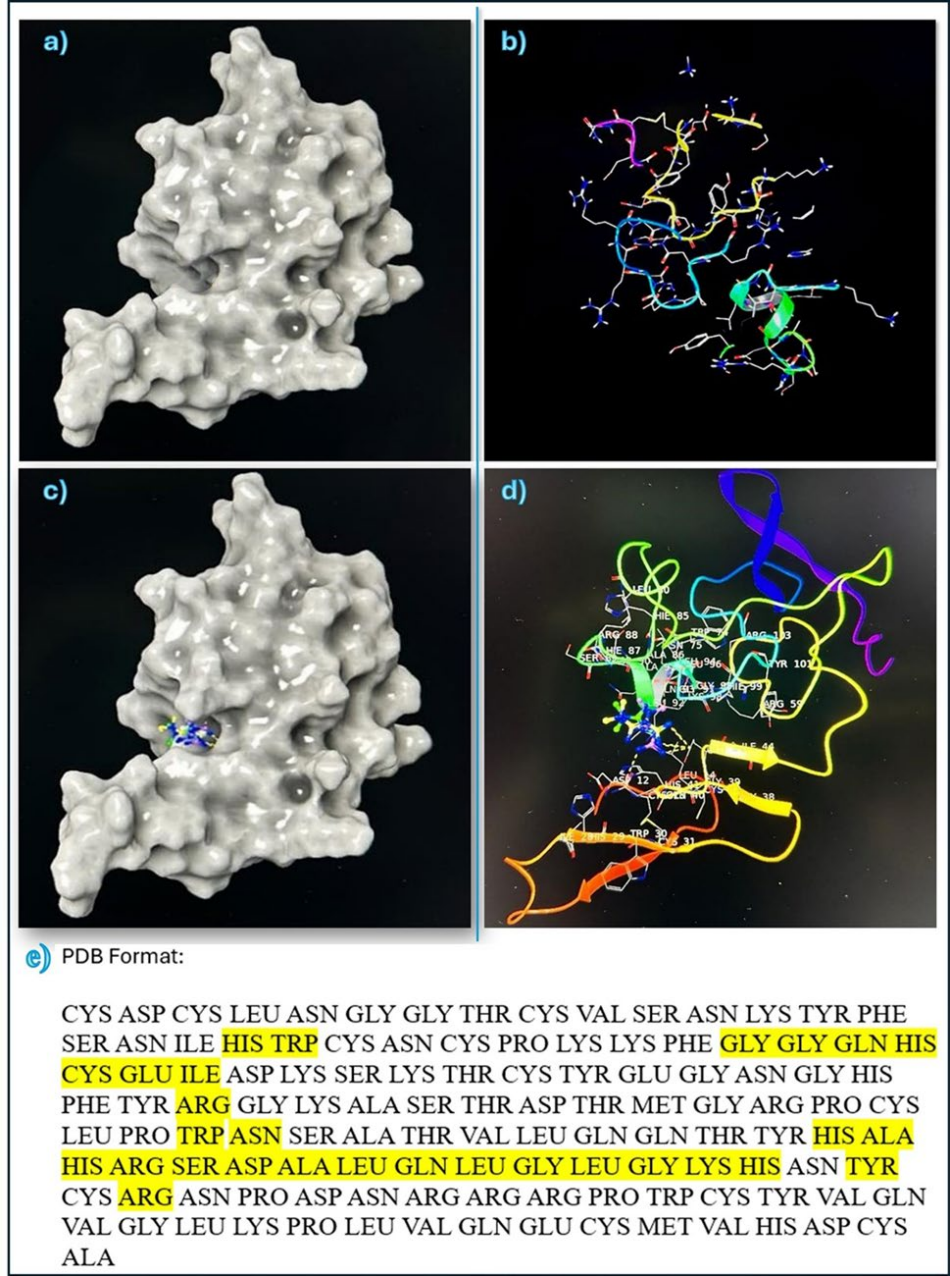
Interaction of uPA protein with Metformin. **a** and **b** show uPA protein structure. **c** and **d** show the 3 poses of metformin in hydrophobic pocket of uPA protein. **e** shows the yellow highlighted amino acids which are the targets of metformin molecule 3 poses in the hydrophobic pocket of chain A in uPA protein by molecular docking of Schrodinger suite version2023−3

**Fig. 8:**
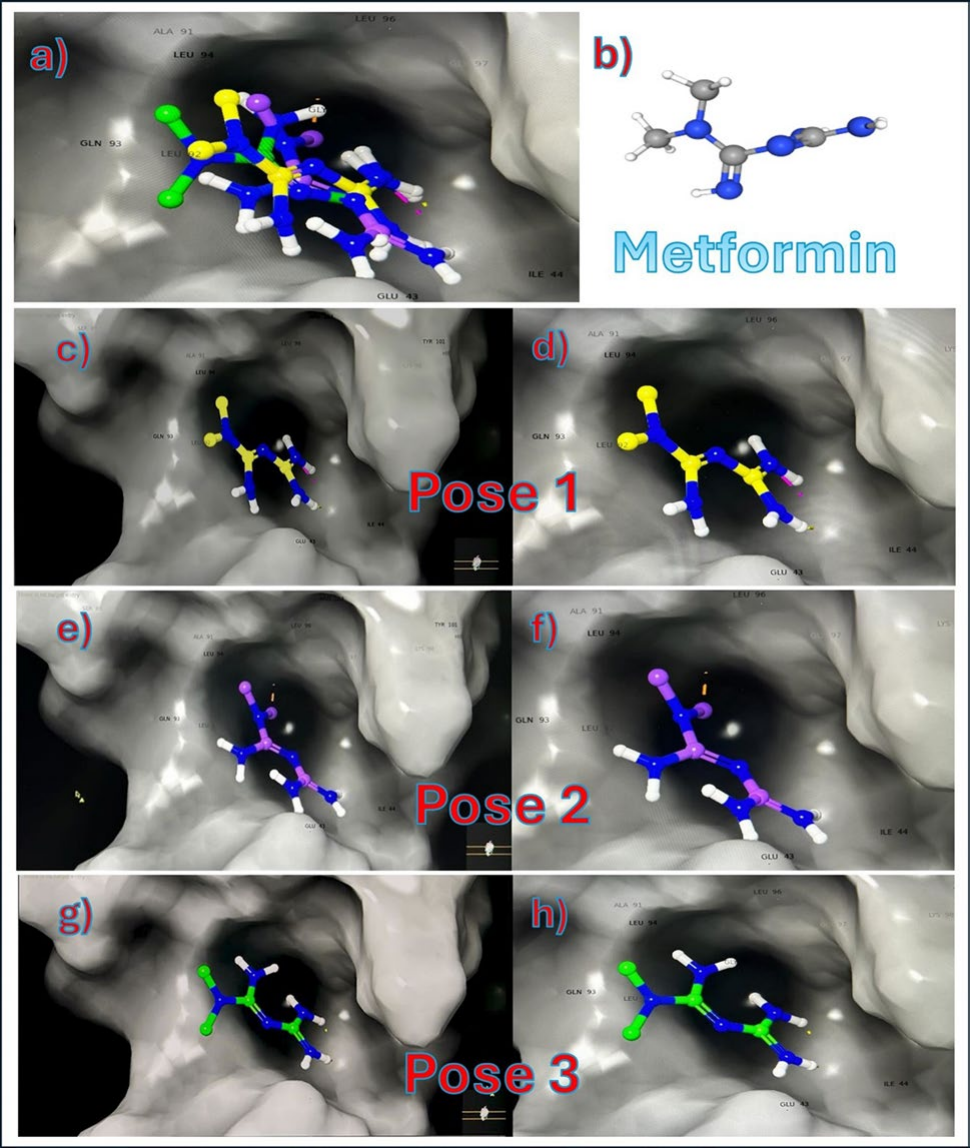
Metformin conformations with uPA protein. **a** shows all 3 poses of metformin combined in the hydrophobic pocket. **b** shows the structure of metformin in 3D. **c** and **d** show pose 1 only in hydrophobic pocket. **e** and **f** show pose 2 only in hydrophobic pocket. **g** and **h** show pose 3 only in hydrophobic pocket

There is a single hydrophobic pocket in which the 3 poses of metformin can interact in a different conformation. The docking results are shown in figures Figs. 7 and 8, showing the uPA protein alone, uPA protein and the 3 poses of metformin together in the same interacting pocket, metformin molecule, and each pose of metformin individually. The amino acids in the hydrophobic pocket, represented by PDB Format, of chain A in which the 3 metformin poses interact with are shown and highlighted in Fig. 7.

#### Molecular dynamic simulation (MDS) of metformin with uPA protein

**Fig. 9:**
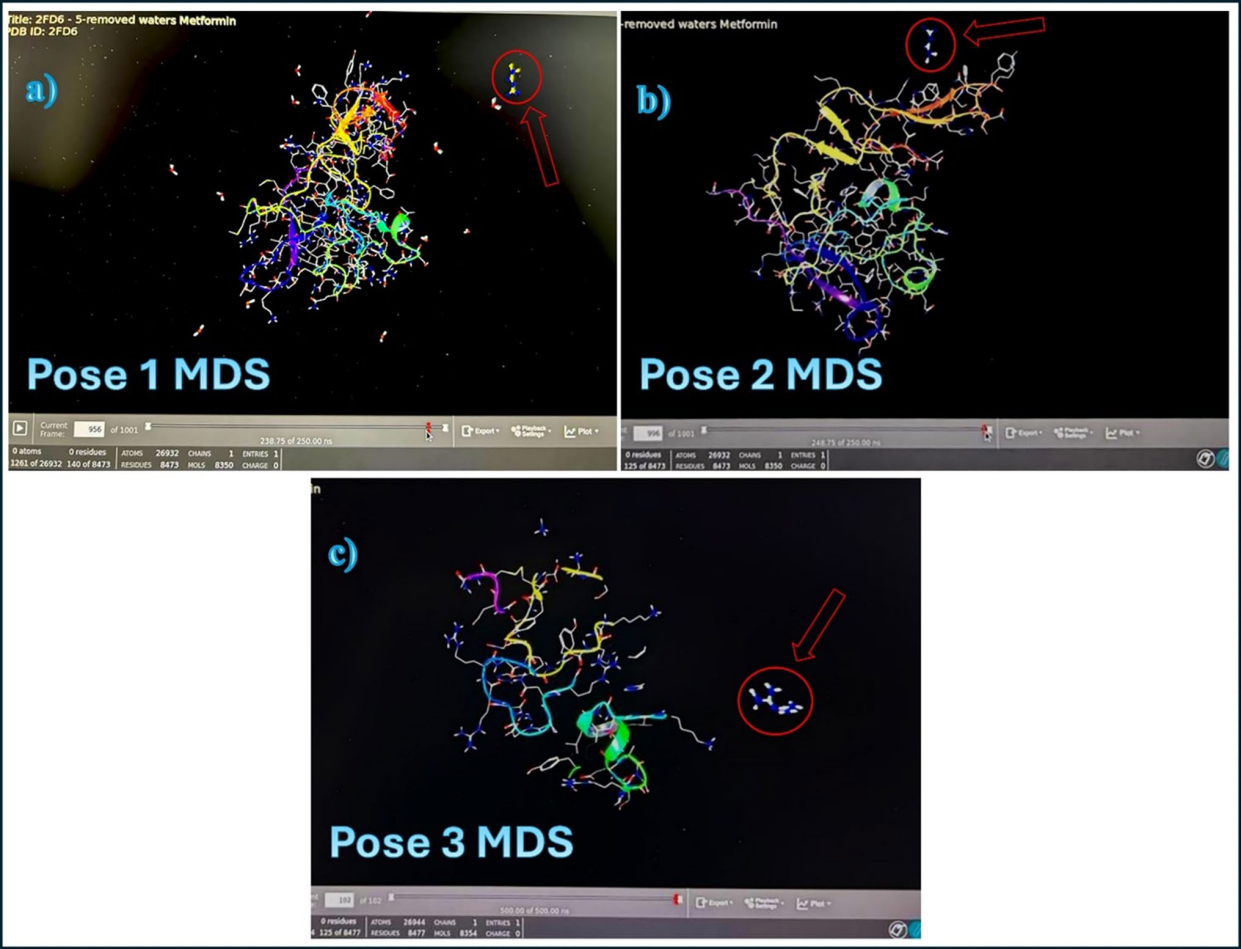
MDS of metformin with uPA protein. **a**, **b**, and **c** are the three poses of interaction of metformin (Arrow) with uPA protein. The stable interaction should last at least 100 ns

The three poses of metformin interaction with uPA protein that were selected in previous step molecular docking are subjected for the MDS to show how far the interaction between metformin and uPA protein can last under normal physiological conditions in a duration of 250 ns (ns). Figure 9 shows the MDS of these poses, respectively, while the videos showing the MDS of each pose are found in the following link (https://drive.google.com/drive/u/0/folders/16XqBJ6IKfPvnYHL_WPxeBq8rMkzlcmFw).

The MDS showed weak interactions of the poses with the uPA protein. The first pose lasted 15.5 ns, the second pose lasted 15 ns, and the third pose lasted only 10 ns. This means that the molecular metformin interaction with uPA protein is very weak and unstable since a stable complex should last at least 100 ns in MDS.

### Experimental validation

#### Metformin cytotoxicity in MDA-MB-231 human breast cancer cell line

Metformin was added in concentrations of 1000, 3000, 5000, 7000, and 9000 µM in triplicate cultures and incubated for 48 h with MDA-MB-231 cell line, and the results show an inhibition of cell viability whenever the concentration of metformin increases, which might help to confirm the inhibition of cell viability by metformin.

**Fig. 10:**
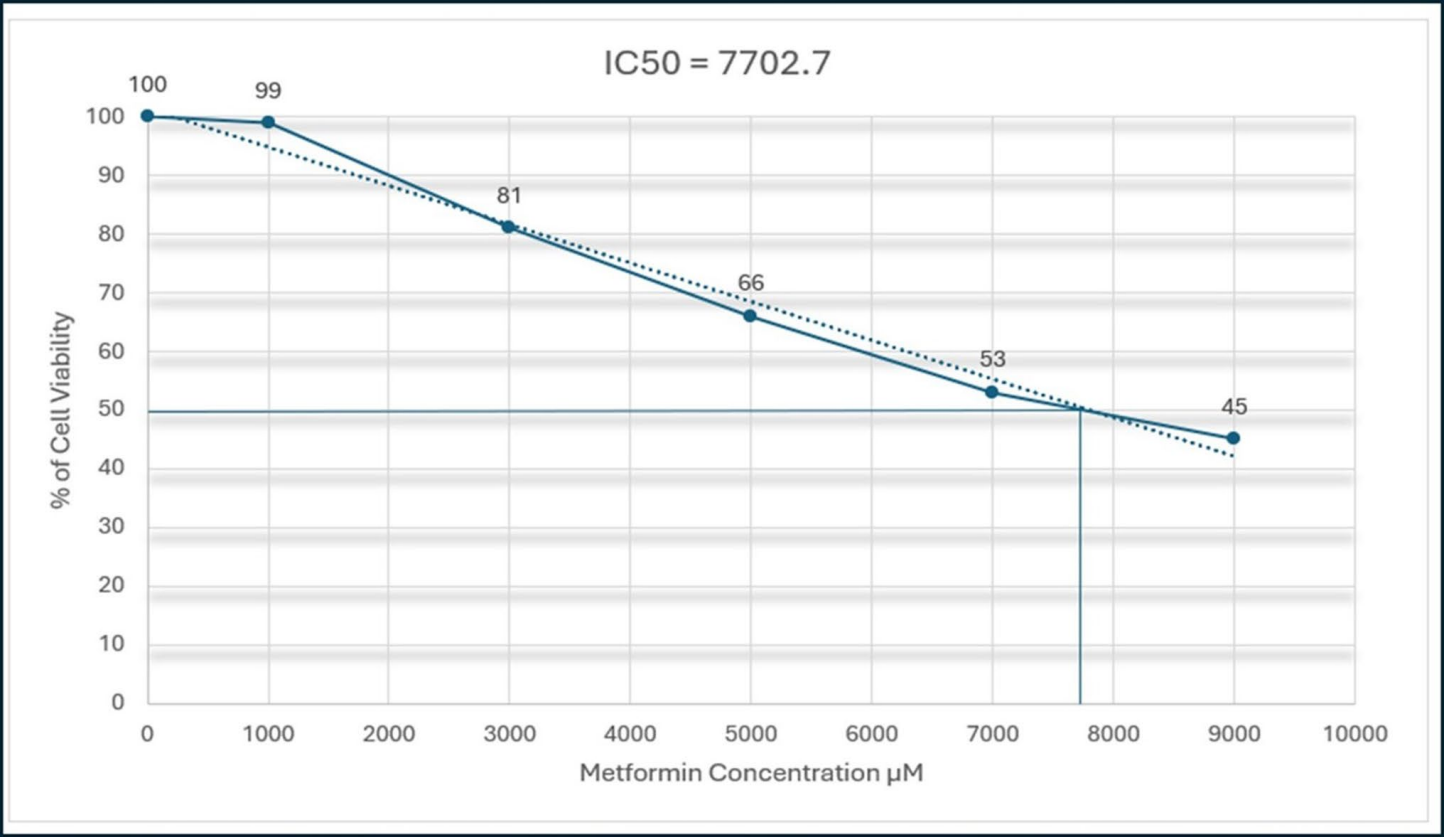
Metformin cytotoxicity in MDA−MB−231 cancer cell line. Metformin was incubated in cells for 48 h, and % of cell viability was presented as mean ± SD of triplicate cultures using MTT Assay

After insertion of the results of the experiment, the IC50 Calculator generates the result in both ways, mathematically and graphically shown in Fig. 10. The results have shown that the IC50 generated is equal to 7702.7 µM.

#### Effect of Metformin on uPA gene expression in MBA-MD-231 human breast cancer cells

**Fig. 11:**
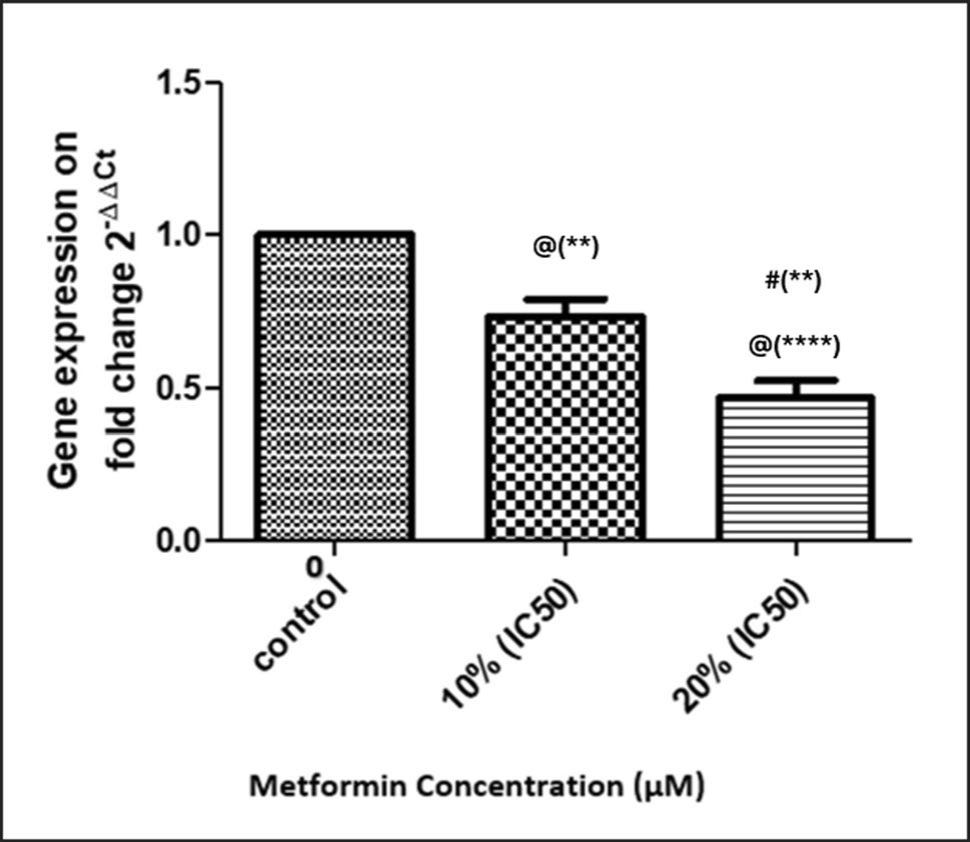
Effect of Metformin on uPA gene expression. Metformin was added in concentrations of 0 (Control), 10% of its IC50 (770.2 µM), and 20% of IC50 (1540.4 µM) in triplicate cultures, and data presented as mean ± SD. Statistical analysis was done by One Way ANOVA and Tukey Kramer as Post ANOVA test. Significance was accepted at *p* ≤ 0.05. @ Significantly different from control. # Significantly different from 10% of IC50. (**p−value < 0.01; ****p−value < 0.0001)

**Fig. 12:**
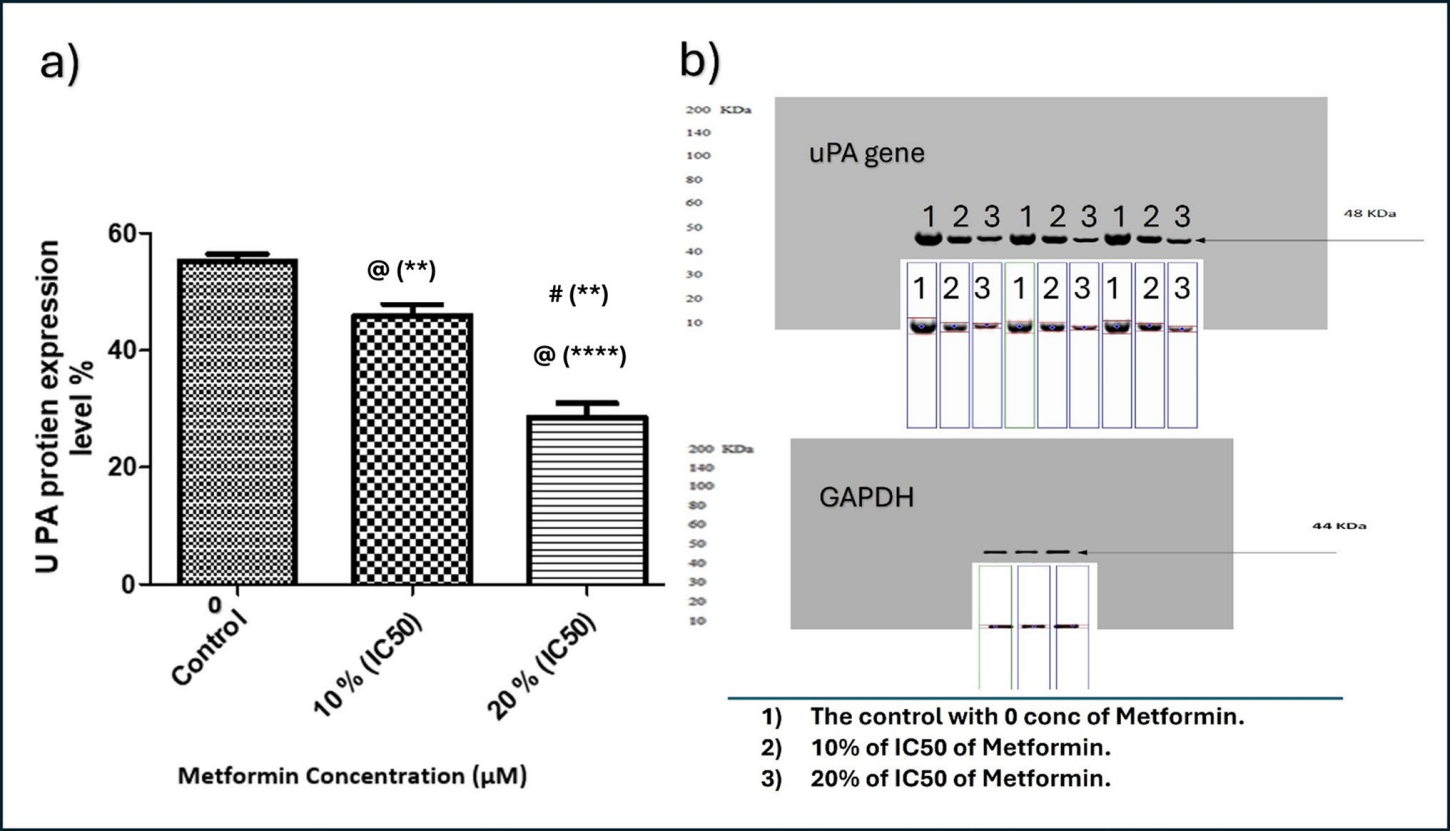
Effect of Metformin on uPA protein content in culture media of MDA−MB−231. Metformin was added in concentrations of 0 (Control), 10% of its IC50 (770.2 µM), and 20% of IC50 (1540.4 µM) in triplicate cultures, and data presented as mean ± SD. **a** % of uPA protein expression. **b** Western Plot of uPA. Statistical analysis was done by One Way ANOVA and Tukey Kramer as Post ANOVA test. Significance was accepted at *p* ≤ 0.05. @ Significantly different from control. # Significantly different from 10% of IC50. (**p−value < 0.01; ****p−value < 0.0001)

As demonstrated in Figs. 11 and 12, treatment of MBA-MD-231 human breast cancer cells with metformin inhibited uPA expression on mRNA Fig. 11 and protein Fig. 12 levels as compared to vehicle-treated cells in a dose-dependent manner.

## Discussion

Metformin, primarily an antidiabetic drug for type 2 diabetes [47], exhibits anticancer activity against tumors such as hepatocellular carcinoma, glioma, lung, and breast cancers [48–51]. Its antitumor effects are mediated by AMPK activation, mTOR inhibition, suppression of insulin-mediated gluconeogenesis, and improved insulin sensitivity, which reduce cancer-promoting metabolic conditions [52, 53]. Additionally, metformin suppresses metastasis and invasion in breast, hepatocellular, and lung cancers [50, 54, 55].

The Tumor Microenvironment (TME) is a complex network of extracellular matrix components and immune cells that infiltrate tumors [25]. Boosting the infiltration of antitumor immune cells represents a promising therapeutic strategy [56]. Drug transporters within TME membranes are critical for drug efficacy, as they enable therapeutic agents like metformin (transported by OCTs) to reach effective concentrations at target sites [57, 58]. These transporters may also act as"gates"to enhance immune cell infiltration, an emerging concept warranting further exploration.

Investigating metformin and its OCT transporters’ impact on the tumor microenvironment (TME) and immune cell activation is an emerging research focus. This study aims to identify optimal tumor targets for metformin to enhance infiltration of clinically favorable immune cells into the TME. Using the TIMER2.0 bioinformatics tool, we analyze metformin/OCT transporter genes (OCT1, OCT2, OCT3) and their correlation with 14 immune cell types (CD8+ T cells, CD4+ T cells, B cells, T cell follicular helper, T cell gamma delta, M0/M1/M2 macrophages, mast cells, neutrophils, dendritic cells, Natural Killer cells, T regulatory cell, monocytes) across 32 TCGA tumor types.

TIMER2.0 has 3 principal components, which are Immune Association, Cancer Exploration, and Immune Estimation. Each of which has its own modules that analyze the complex interactions among immune infiltrates, genes of interest, clinical outcome of immune infiltrates and/or genes of interest, chances of mutation of the genes of interest in different tumors, and differences in gene expression rates between adjacent normal tissues and malignant cells in the TME; moreover, each of these modules use different algorithms to reach the intended results [32].

In the present study, Gene Module of Immune Association Component investigates the correlation between OCT1, OCT2, and OCT3 genes and the 14 immune cells of interest in 32 types of cancer of TCGA data [32]. Results revealed that immune infiltrates that show the highest number of positive correlations with OCT1, OCT2, and OCT3 genes are T regulatory cells, Macrophage M1, Bcell, Mast Cell, and Macrophage M0. While the tumor types that showed the highest number of positive correlations with OCT1, OCT2, and OCT3 genes are BRCA, LIHC, LUSC, SKCM-Metastasis, SKCM, THYM, KIRC, and LUAD.

It's known that a low tumor purity, proportion of tumor cells in the TME in comparison to non-tumor cells, sample indicates higher amount of immune infiltrates in TME; as a result, immune cells'inflammatory reaction occur, which may enhance the efficacy of natural immunity against tumor [59]. As a result, we managed to seek the tumor purity of the tumor types obtained from Gene Module in Immune Association Component to correlate between the presence of immune infiltrates and the tumor purity results that might highlight the prognosis of the tumor types investigated. The results show that BRCA, BRCA-LumA, OV, THYM, BLCA, and LUSC had negative tumor purity result (Rho < 0) with OCT1, OCT2, and OCT3 all together, referring to that immune cells in these types of tumor might show greater prevalence in TME over the tumor cells and thus better prognosis.

Furthermore, Outcome Module of Immune Association Component studies the clinical impact of all 14 types of immune infiltrates on patient survival in different 32 tumor types of TCGA data [32]. Results revealed that the immune infiltrates that showed the highest frequency of decrease risk are CD8+ Tcell, Macrophage M1, and Tcell Follicular Helper. CD8+ Tcell showed decrease risk in SKCM, HNSC, LIHC, BLCA, DLBC, BRCA, SKCM-Primary, SKCM-Metastasis, CESC, and HNSC HPV+ve. Macrophage M1 showed decrease risk in SKCM, BRCA, BRCA-LumA, BRCA-LumB, OV, and SKCM-Metastasis, and Tcell Follicular Helper showed decrease risk in STAD, THCA, OV, BRCA, and BRCA-LumA. Decrease risk result indicates that the immune infiltrate has good clinical outcome through the attack of immune infiltrates on the tumor to yield better prognosis [32]. On the other hand, the immune infiltrate that shows the highest frequency of increase risk is Macrophage M2 that has been already reported that is has a protumor effect and it can promote tumor progression [60].

To identify the immune infiltrate that is positively correlated to OCT genes and has decrease risk (good clinical outcome) and found in most of the tumor types that show positive correlation with OCT genes, an intersection analysis should be done for the results obtained in both Gene Module and Outcome Module. This intersection might be effective by detecting the immune infiltrates that were highly infiltrated into the tumor’s TME to reach the best therapeutic and most prevalent immune infiltrate along with the best type of tumor in which this immune infiltrate can show its effect. This intersection is not involved in TIMER2.0 originally [32]; therefore, the intersection is performed by using Venny website which is also another bioinformatic tool specified only for intersections [27].

The results of the intersection analysis illustrate that Macrophage M1, that has decrease risk effect, has the highest presence along with 4 different tumor types of TCGA data, and BRCA shows the highest presence of immune infiltrates in its TME, among them is the Macrophage M1. This highlights that BRCA is the most promising tumor type that shows the best clinical outcome when OCT genes are provoked by metformin to let immune infiltrates, particularly Macrophage M1, enter the TME. Macrophage M1 is known in different studies as immune infiltrate that attacks tumors and inhibits their progression [60, 61].

Our results are assured by establishing another intersection between the same types of tumors that show positive correlation with OCT1, OCT2, and OCT3 genes and all immune infiltrates that show significant clinical outcome, either increase or decrease risk. Again, the second intersection analysis shows that Macrophage M1 expresses the highest prevalence in all tumor types as decreased risk, and BRCA shows the highest presence of immune infiltrates into its TME, including Macrophage M1.

This important result may indicate that metformin can activate OCT to increase metformin’s transport into TME of BRCA in high concentrations and enhance the infiltration of Macrophage M1 into TME to give chance for both metformin itself and Macrophage M1 to attack the tumor cells of BRCA. Since Macrophage M1 is among the innate immunity, it plays a major role as an antitumor through secretion of Reactive Oxygen Species and Nitric Oxide that directly attack the tumor cells [62]. Therefore, activation of Macrophage M1 and/or increase its infiltration into tumor cells could be considered as possible therapeutic target in treatment of BRCA.

Somatic mutations are important for carcinogenesis and have a significant impact on patients ability to respond to therapy and survive. Furthermore, determining how somatic mutations affect the pattern of gene expression and function is essential for precision oncology, which relies on the identification of genetic drivers of cancer and medication development [63]. In the present study, Mutation Module in TIMER2.0 can examine and observe the impact of non-synonymous somatic mutations of OCT1, OCT2, and OCT3 genes on immune cell infiltration across a variety of cancer cell types [32]. The results illustrated that OCT1, OCT2, and OCT3 genes, represented by gene names SLC22 A1, SLC22 A2, and SLC22 A3 respectively, showed low incidence of mutation rates. This result means that OCT genes are sustainable, consistent, and reliable to use them in further studies as detrimental factors for gene application and in targeting these genes in therapy [64, 65].

In Cancer Exploration Component in TIMER2.0, Gene_DE Module is able to compare between the status of OCT genes either upregulated or downregulated in normal tissues surrounding tumors and their status in tumor tissues in 32 tumor types of TCGA data [32]. In this section, although the comparison is held in all 32 types of tumors in TCGA data, we focused on BRCA as it showed the most suitable target for metformin. The results showed that OCT1 and OCT3 are more expressed in BRCA tissue more than they are in normal breast tissue, while OCT2 showed no significant difference in normal breast tissue in comparison to BRCA tissue. This assures that OCT1 and OCT3 genes expression may allow higher concentrations of metformin and Macrophage M1 in BRCA tissue than they would in normal tissue, all increasing their chances for their therapeutic antitumor effect. Enhancing drug delivery to target tissue is considered one of the important tasks in cancer therapy [25, 66].

In the same component, Gene_Outcome Module in TIMER2.0 provides the clinical relevance of OCT1. OCT2, and OCT3 genes to investigate the clinical outcome of the expression of OCT genes as an individual, independent factor particularly in BRCA. The analysis revealed that only OCT2 gene expression showed a significant decrease risk result in BRCA, while OCT1 and OCT3 gene expression showed non-significant effect.

In another point of view, tumors secrete several proteins in TME to help itself in metastasis, angiogenesis, and invasion [67]. Yong P Hwang *et.al.* [68]*,* reported that metformin has an effect on HT-1080 cell by prohibiting its invasion and metastasis through the inhibition of MMP-9 protein and gene expression. In another study by Hsieh *et.al*, [69] metformin inhibited the invasion of hepatocellular carcinoma through the inhibition of uPA and MMP-9 expression. These data stimulated our interest to investigate the possible interactions of metformin with human proteins that are produced by the tumor in TME. Therefore, a bioinformatic tool (SwissTargetPrediction) was used, which enables to predict the possible interaction of metformin with possible human target proteins. The program revealed that metformin can interact with several target proteins, among them is uPA which is of interest due to its marked involvement in tumor metastasis [70]. The prediction result is revalidated by using another bioinformatic prediction tool (Way2 drug), which also shows a probability of interaction between metformin and uPA protein. Targeting oncoproteins is essential therapeutic strategy in treatment of cancer [71]. As it is also known, uPA is linked with poor prognosis in different types of tumors such as prostate cancer [72], breast cancer [73], liver cancer [74], ovarian cancer [75], and gastric cancer [76]. These studies encourage us to investigate the possible interactions of metformin with uPA protein and/or gene expression.

To study the possible interactions of a drug and a target protein, there are 3 efficient ways through which these interactions could be examined: direct chemical interaction to neutralize the protein, inhibition of protein’s gene expression, or inhibition of protein’s transcription. In the present study, computational analysis was conducted to highlight the possible interactions between metformin and uPA protein using molecular docking. The results revealed that metformin can interact with uPA protein in 3 different poses but all at the same hydrophobic pocket in the protein.

To test the stability of these complexes, Molecular Dynamic Simulation (MDS) technique has been conducted to evaluate the 3 poses of metformin-uPA protein complex. Results of MDS showed that metformin-uPA protein complex is not stable; in other words, it seems that the chemical interaction of metformin with uPA protein is not apparently the way that metformin interacts with uPA protein.

To assess the sustainability of the results obtained, Mutation Module of Immune Association Component of TIMER2.0 has been used to analyze the frequency of mutation of uPA gene. The results showed that, among the different 32 types of tumor, uPA gene had very low mutation frequency in BRCA, which emphasized the credibility and sustainability of the results obtained.

Therefore, it is crucial to prove the effect of metformin on BRCA and uPA, experimentally, to validate all the previous results collected from bioinformatic tools. To investigate the effects of metformin, MDA-MB-231 cell line was used, which is a type of breast cell lines that expresses uPAR/uPA genes highly [77], and it was used to test the cytotoxicity of metformin and identify its IC50. Additionally, the effect of metformin on uPA gene transcription and gene translation was undergone by using qRT-PCR and Western Blot, respectively.

The results revealed that metformin, in subtoxic concentrations of 10% and 20% of IC50, inhibited uPA gene expression on mRNA and protein levels in MDA-MB-231 cell line in a dose-dependent manner. These effects are supported also in different studies by Lingling Fang *et.al.,* and Yong P Hwang *et.al.,* who showed that metformin inhibited uPA and MMP-9 in esophageal squamous cell carcinoma and human fibrosarcoma HT-1080 cells [68, 69]. Moreover, Hart *et.al.*, [78] reported that metformin doesn’t interact directly with uPA but rather downregulated the protein expression, strongly supporting our findings.

**Fig. 13:**
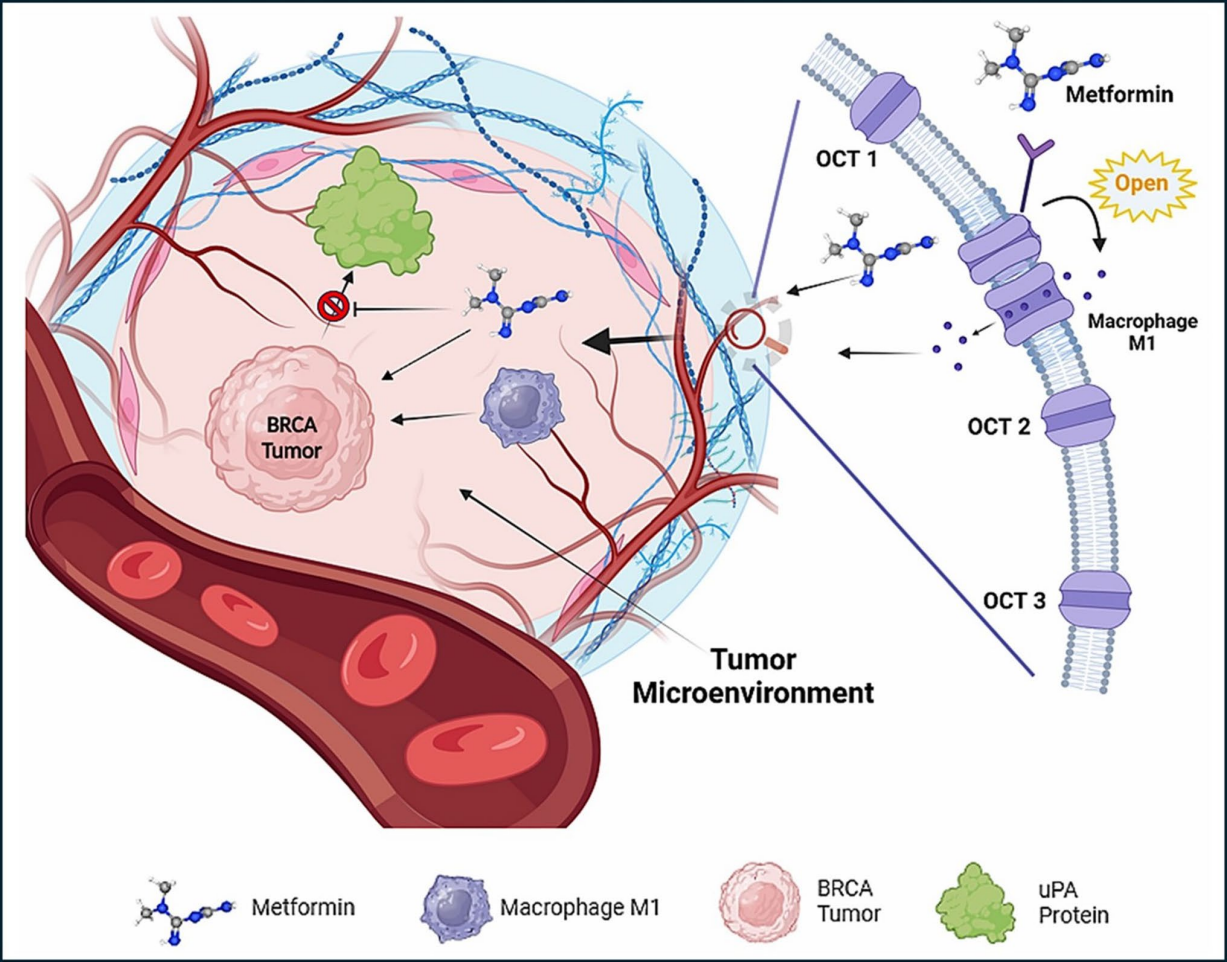
Proposed role of Metformin and its transporters in Tumor Microenvironment

In conclusion, metformin has a possible therapeutic effect on BRCA directly through its cytotoxic effect, indirectly through enhancing the passage of Macrophage M1 immune infiltrate that has a good clinical outcome into the TME, and through inhibiting tumor metastasis through the inhibition of uPA gene expression and translation, Fig. 13.

### Study limitations

1. MDA-MB-231 cell line is used in this study due to several reasons; this cell line is triple negative (negative Estrogen, Progesterone, and HER2 receptors), and has strong epithelial-to-mesenchymal transition (EMT) features which are associated with increased motility, invasiveness, and metastatic potential. Moreover, it has a drug resistance capacity, and it highly expresses uPA protein, which is the protein target in this study [79, 80]. However, it’s still a limitation to use one cell line that could be resolved by using other additional cell lines in the future to provide more reliable and reproducible data.
2. In this study, OCT1 and OCT3 showed upregulation along with Macrophage M1 infiltration in BRCA as correlative relationship. It would be more beneficial if cause-effect relationships were investigated in a separate future study using knockdown study or pharmacologic inhibition of these genes to ensure their activity vs. Macrophage M1 infiltration.

## Supplementary Information

Additional file1

Additional file2

Additional file3

Additional file4

Additional file5

Additional file6

Additional file7

Additional file8

Additional file9

## Data Availability

No datasets were generated or analysed during the current study.
