# Supplementary material for: Modulatory effect of metformin and its transporters on immune infiltration in tumor microenvironment: a bioinformatic study with experimental validation
Source: Discov Oncol. 2025 May 31;16:973. doi: 10.1007/s12672-025-02766-y (PMC12126455; doi:10.1007/s12672-025-02766-y)
Supplement: Supplementary file 3 — Additional file3 [file 12672_2025_2766_MOESM3_ESM.pptx]

## Slide 1
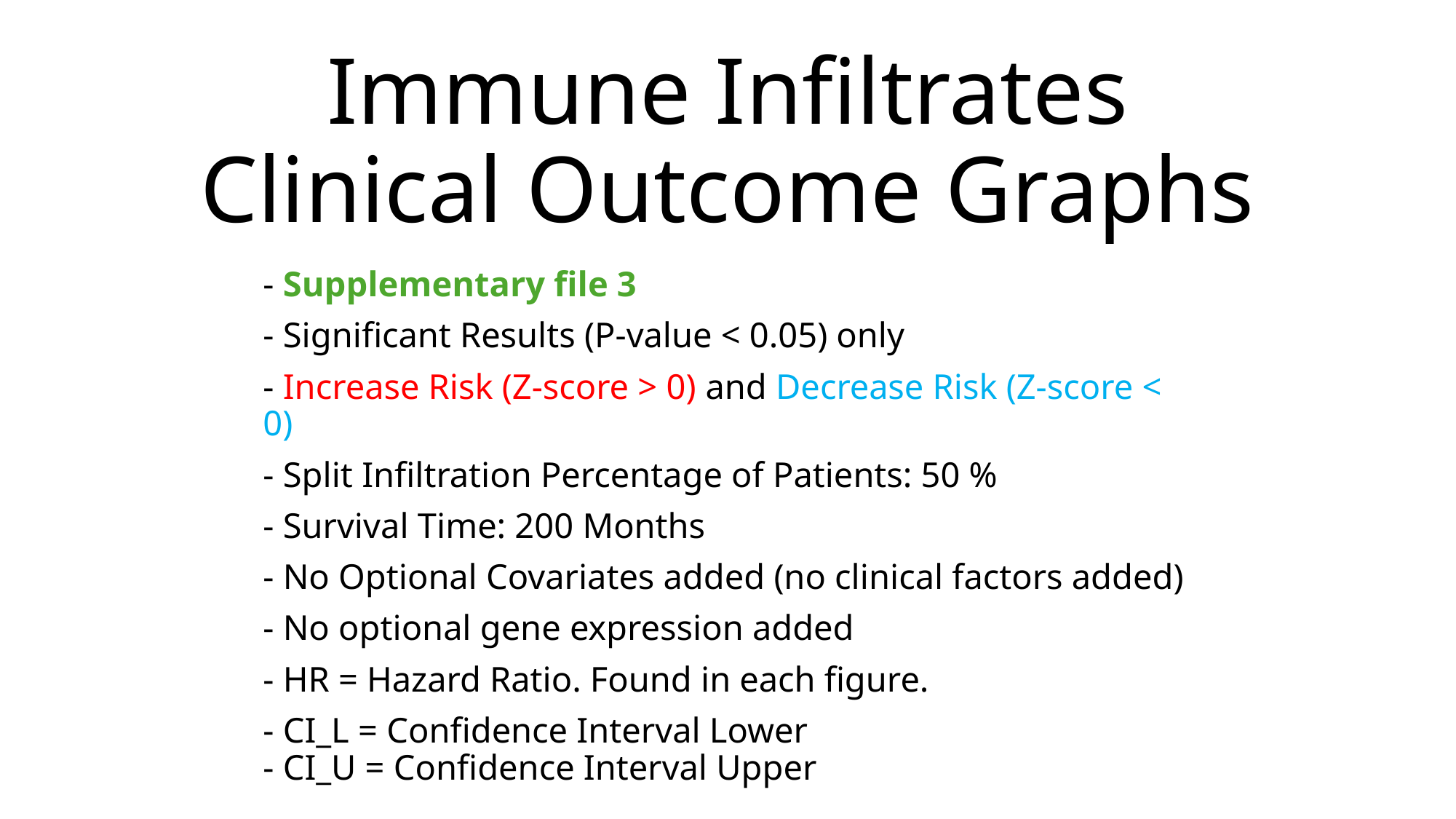

# Immune Infiltrates Clinical Outcome Graphs
- Supplementary file 3
- Significant Results (P-value < 0.05) only
- Increase Risk (Z-score > 0) and Decrease Risk (Z-score < 0)
- Split Infiltration Percentage of Patients: 50 %
- Survival Time: 200 Months
- No Optional Covariates added (no clinical factors added)
- No optional gene expression added
- HR = Hazard Ratio. Found in each figure.
- CI_L = Confidence Interval Lower
- CI_U = Confidence Interval Upper

## Slide 2
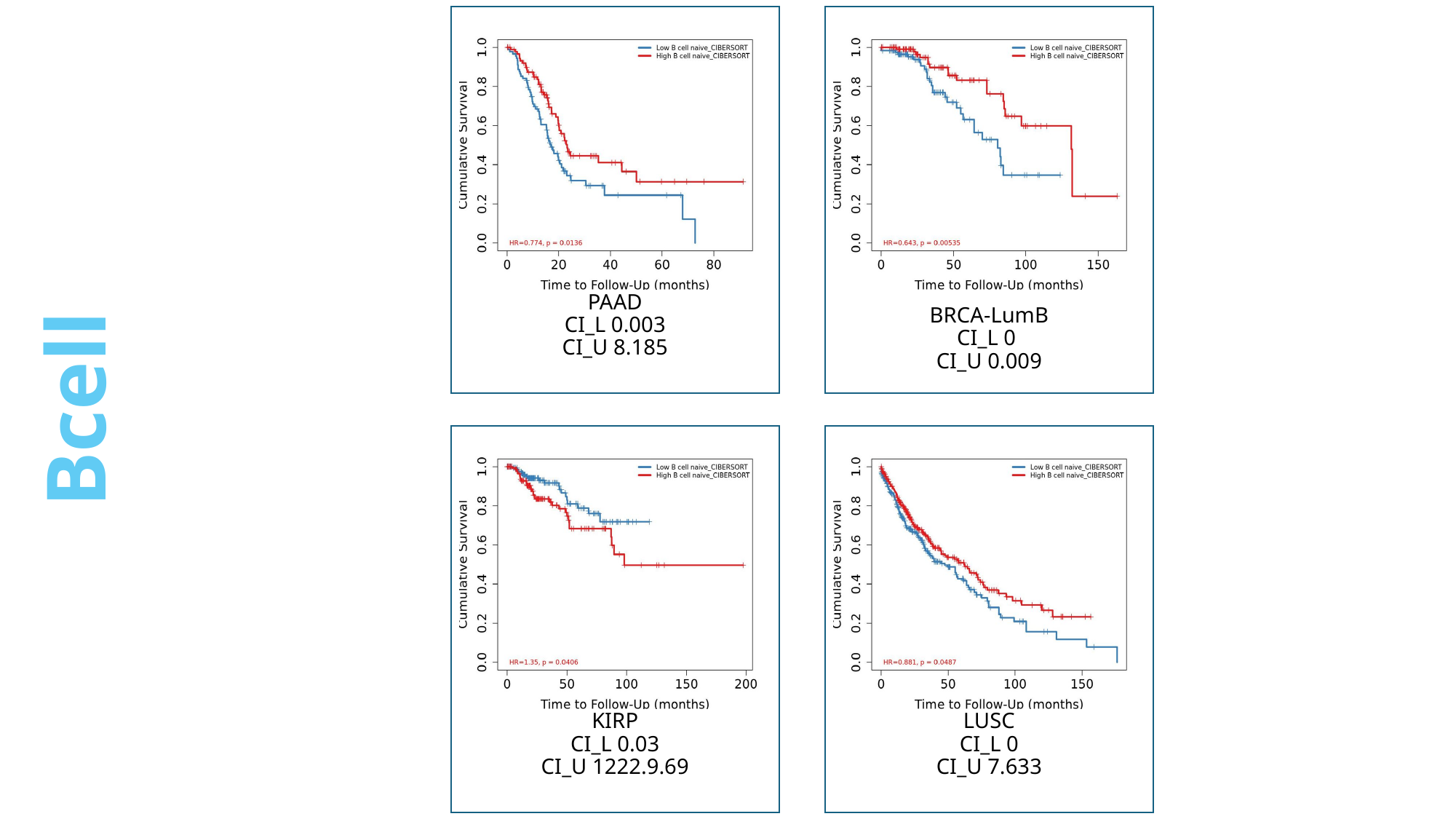

Bcell

## Slide 3
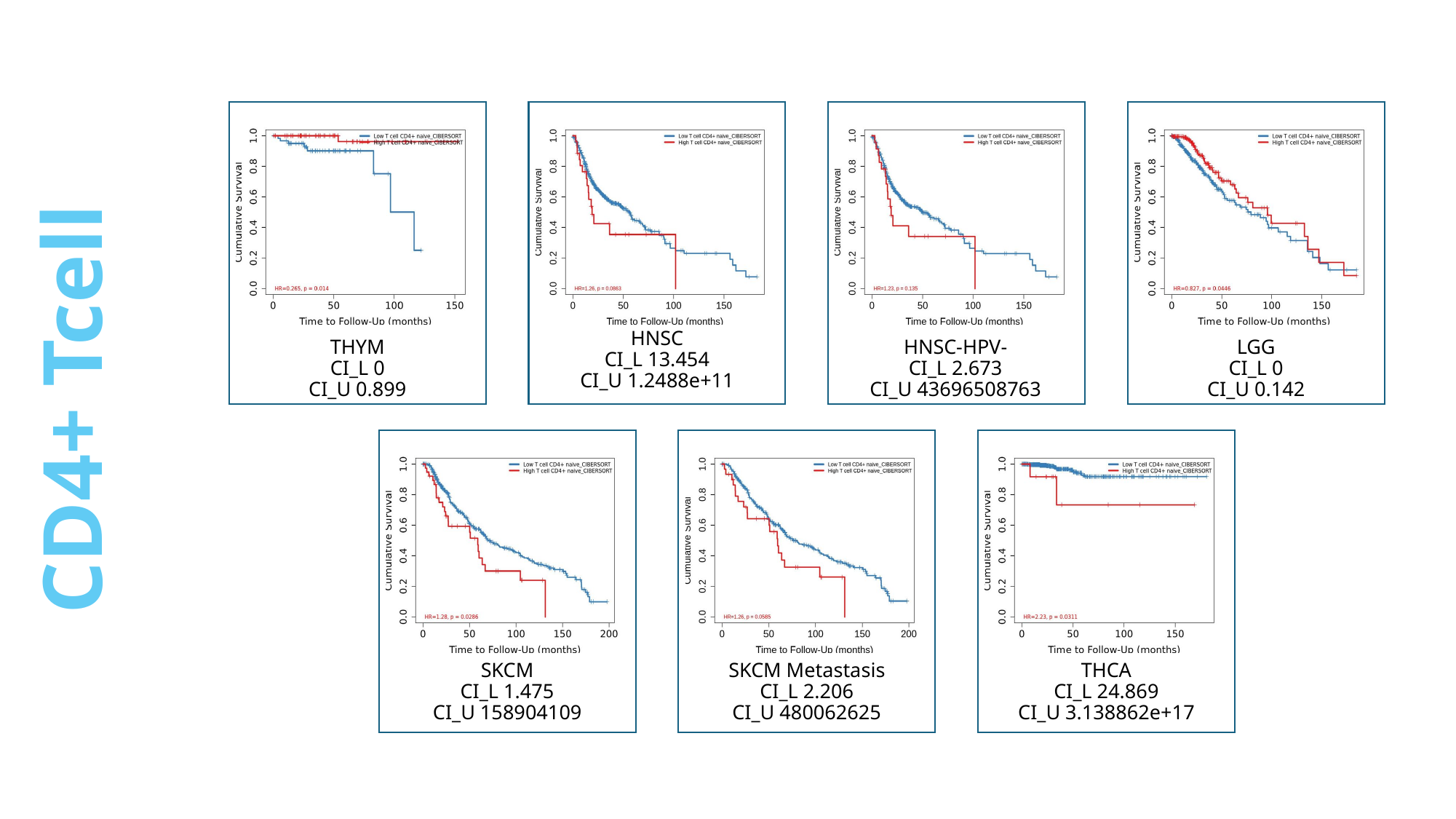

CD4+ Tcell

## Slide 4
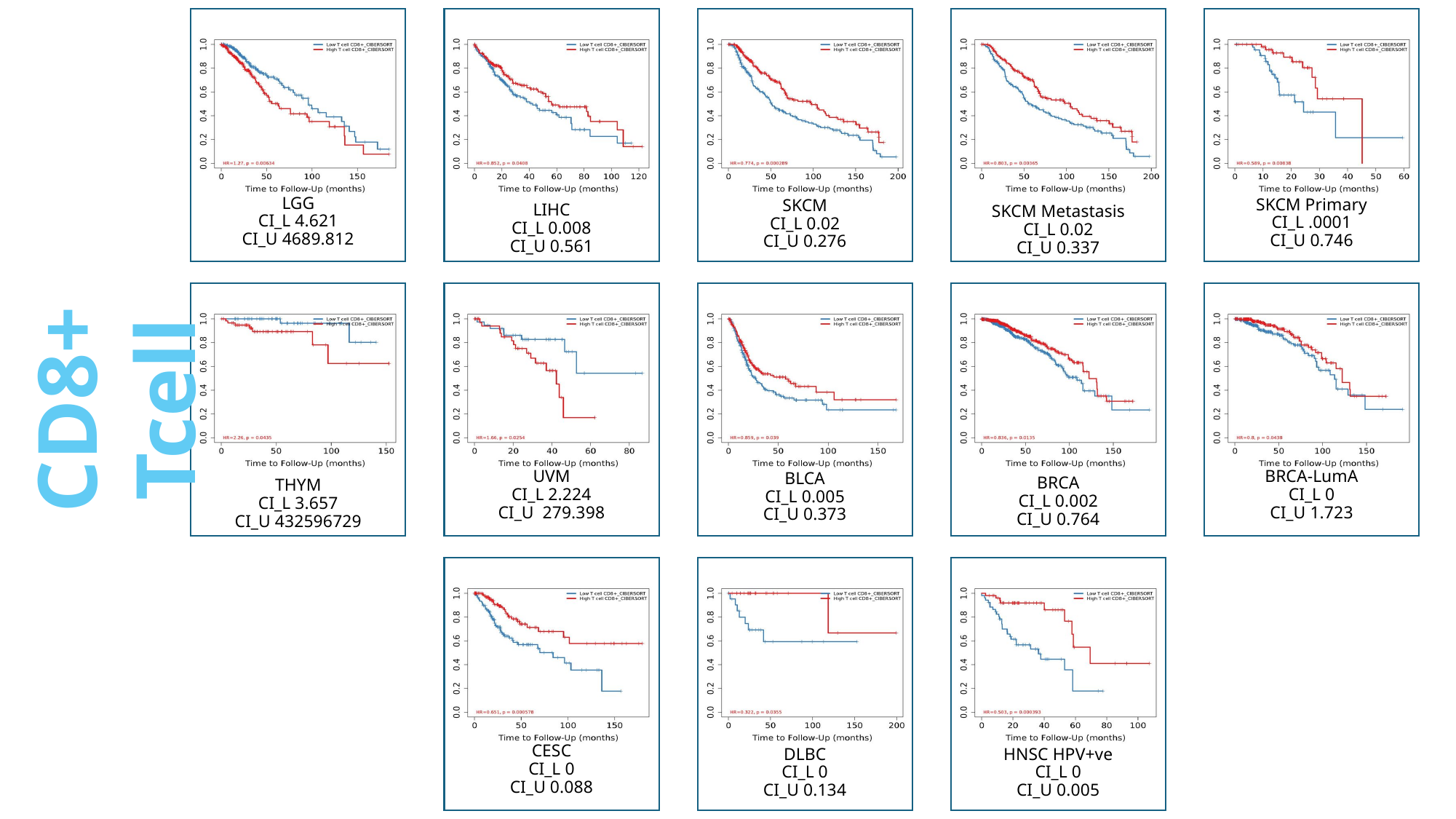

CD8+ Tcell

## Slide 5
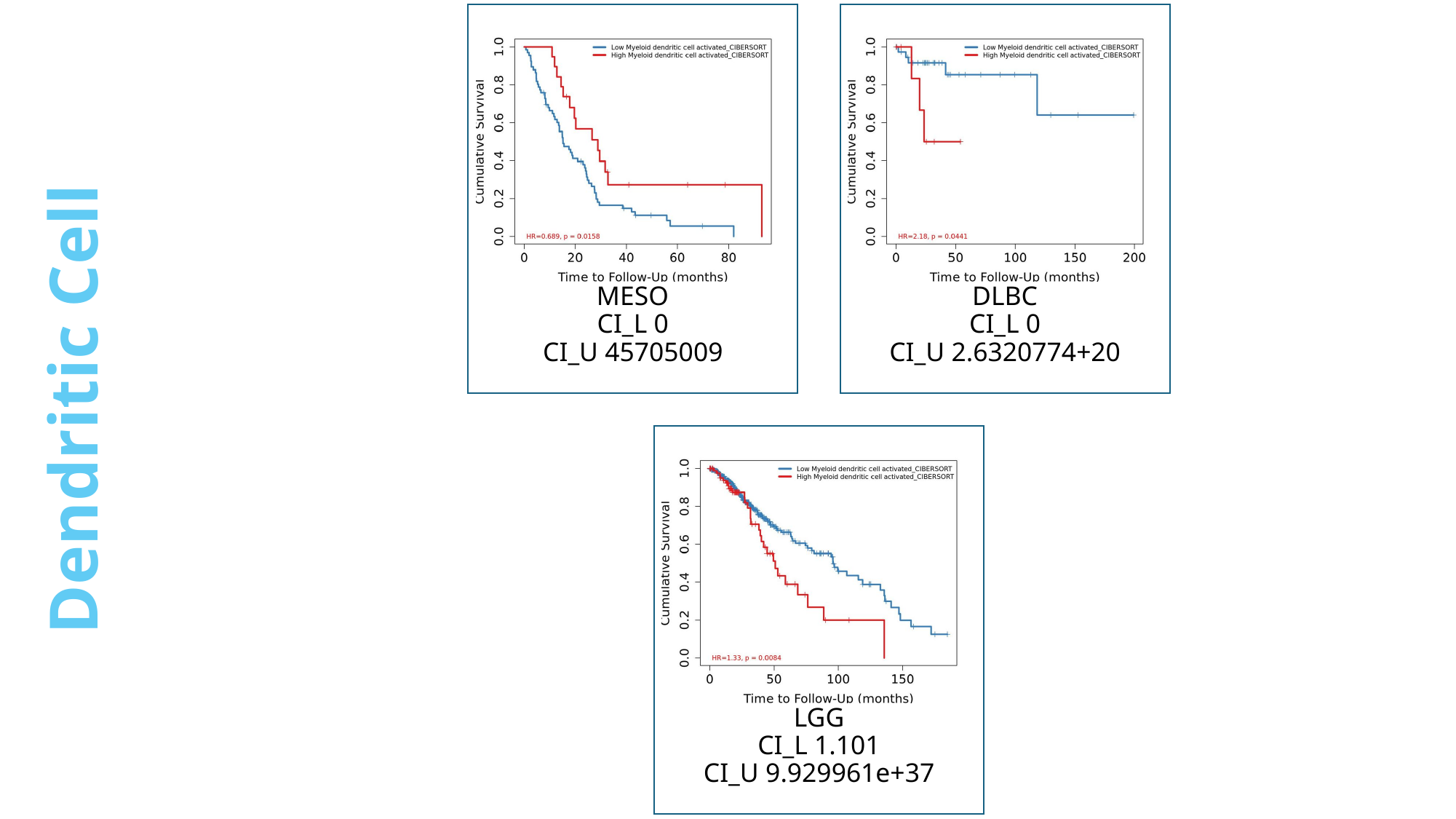

Dendritic Cell

## Slide 6
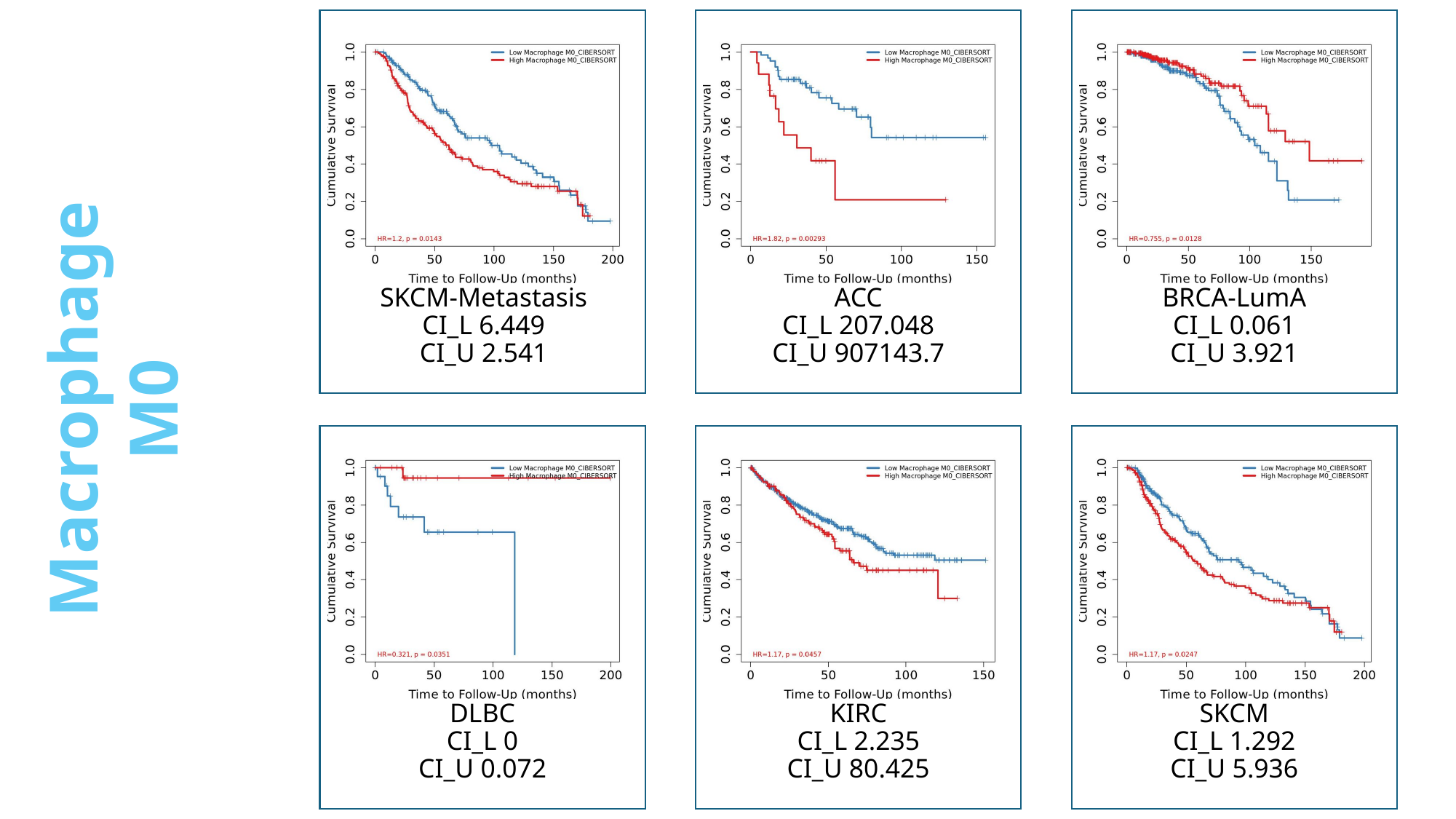

Macrophage M0

## Slide 7
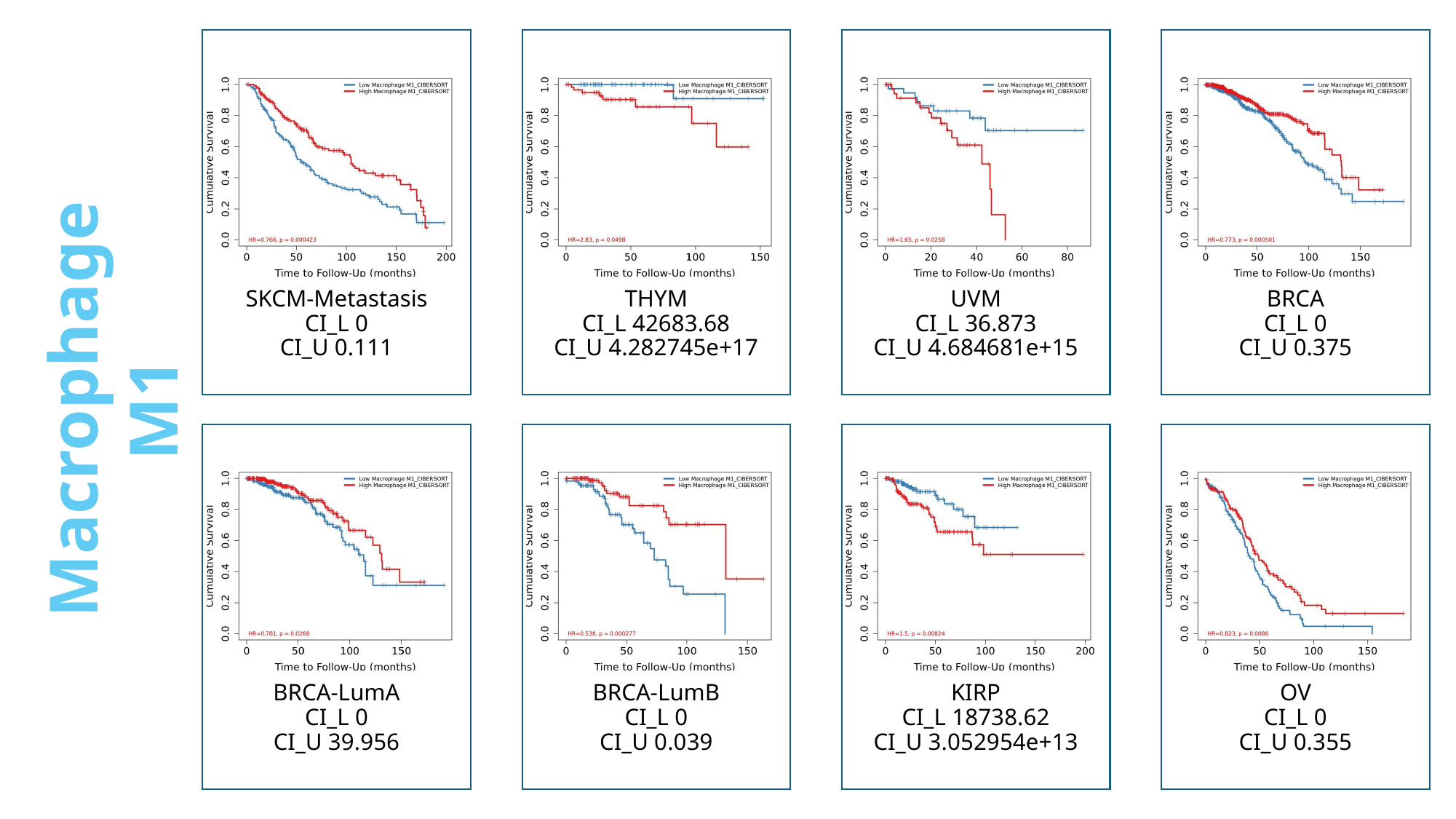

Macrophage M1

## Slide 8
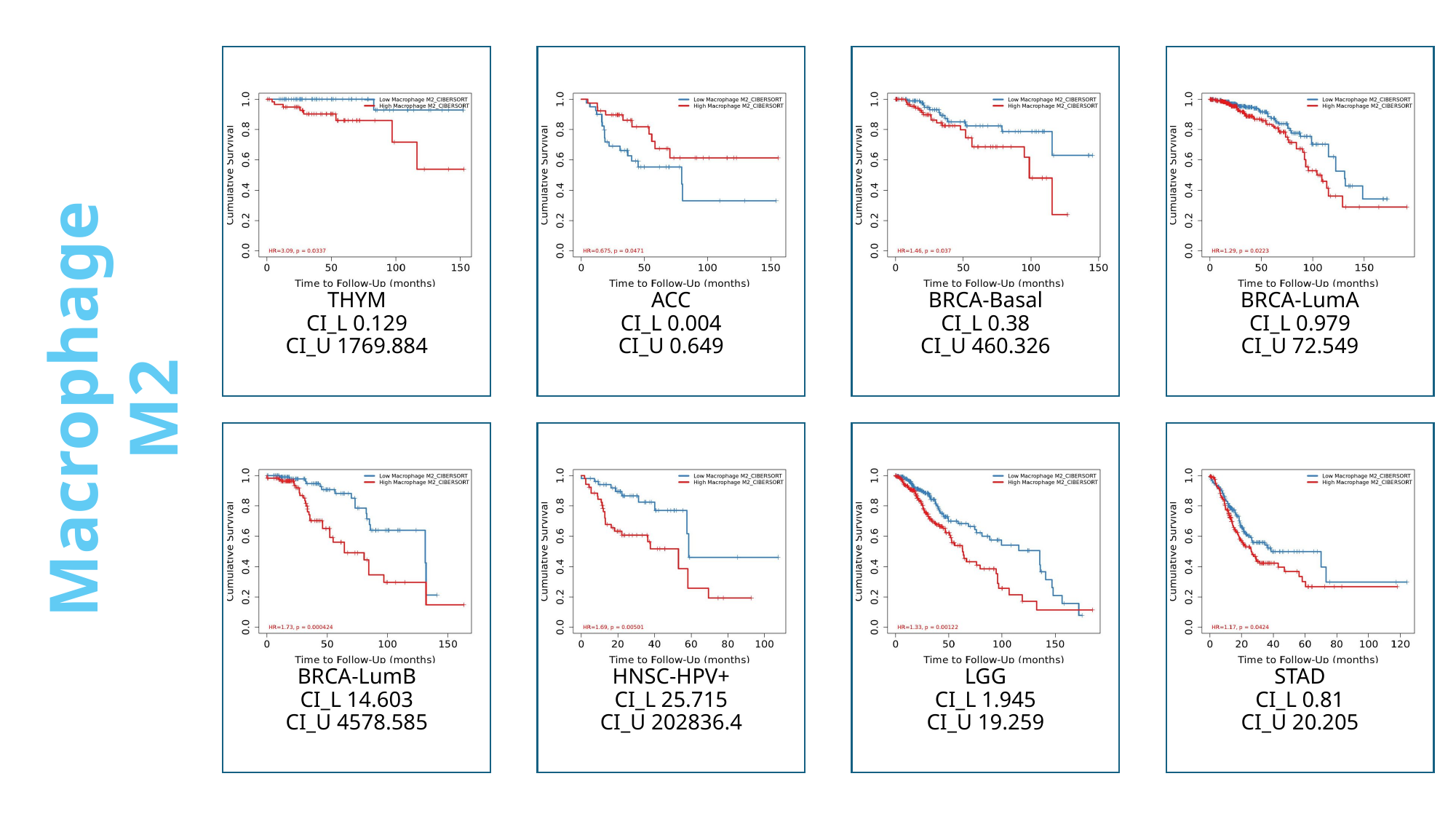

Macrophage M2

## Slide 9
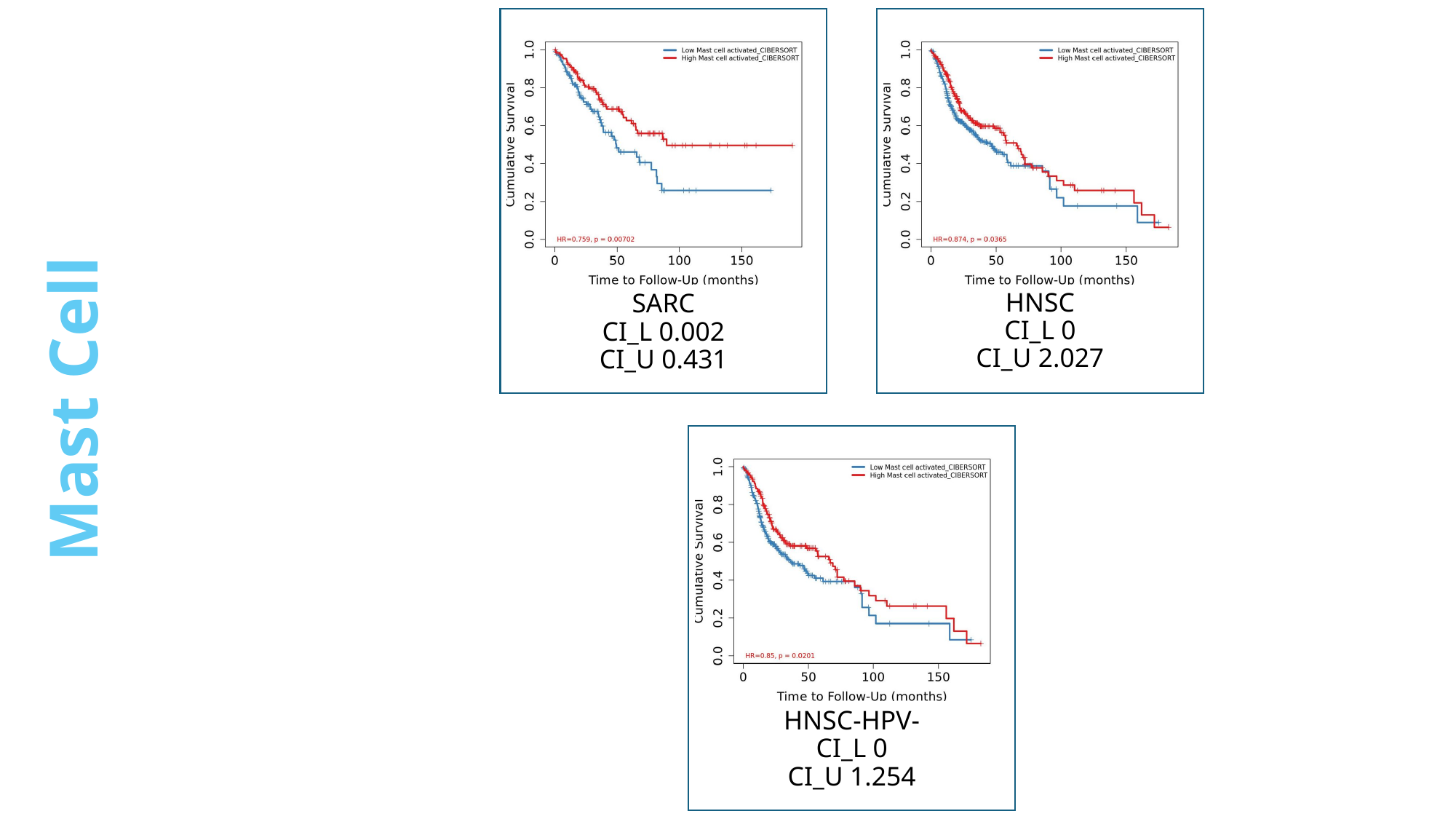

Mast Cell

## Slide 10
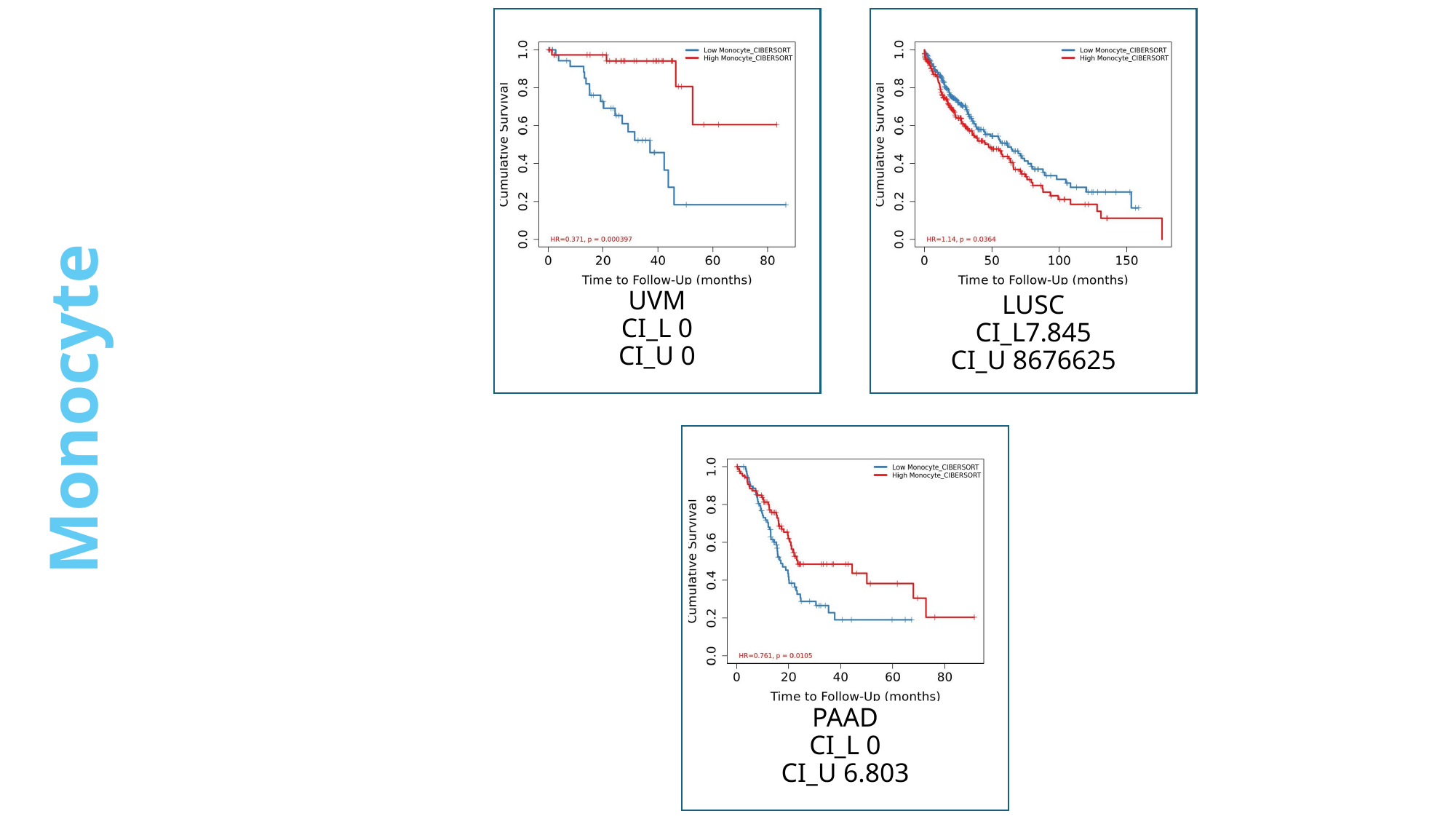

Monocyte

## Slide 11
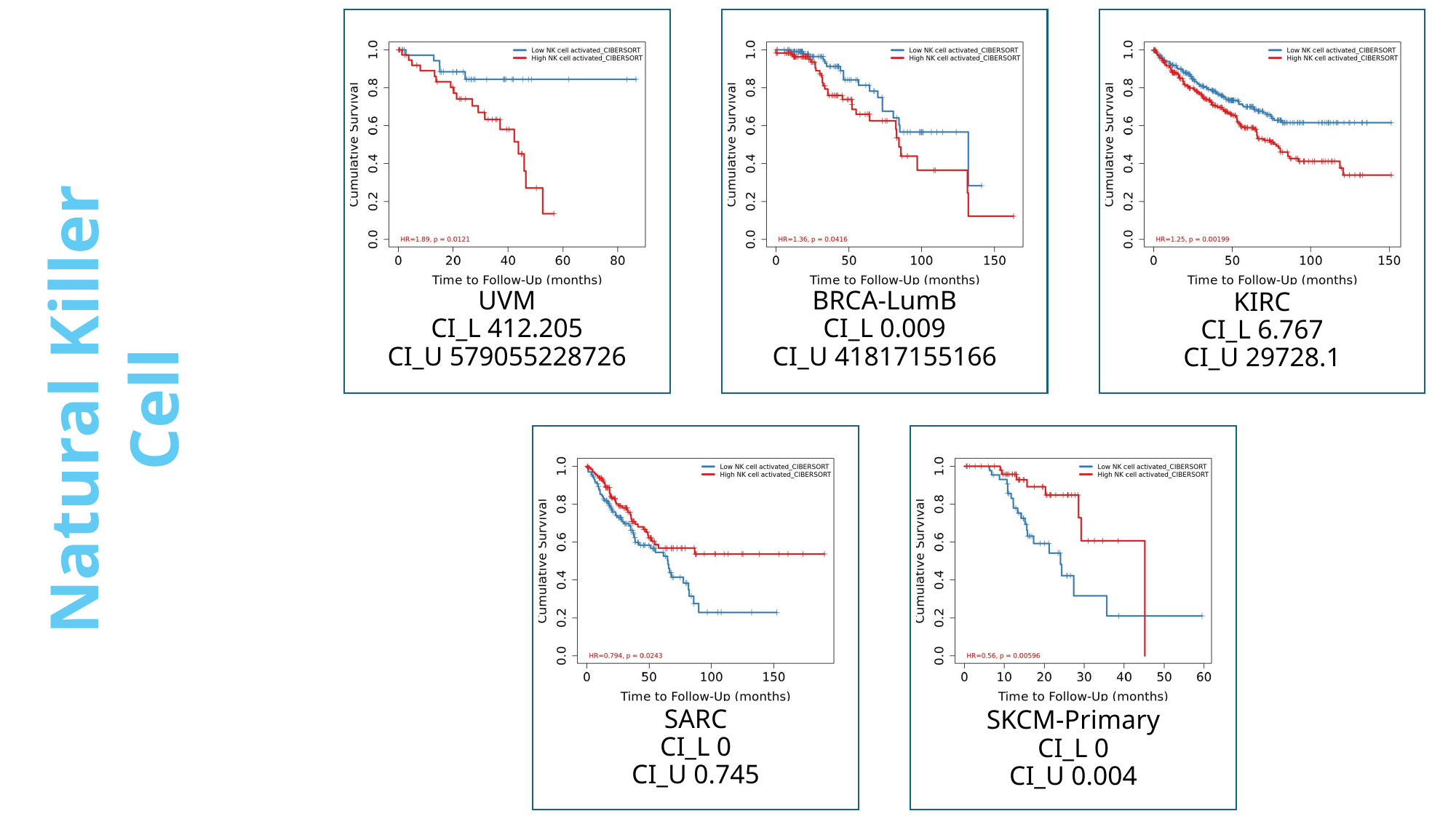

Natural Killer Cell

## Slide 12
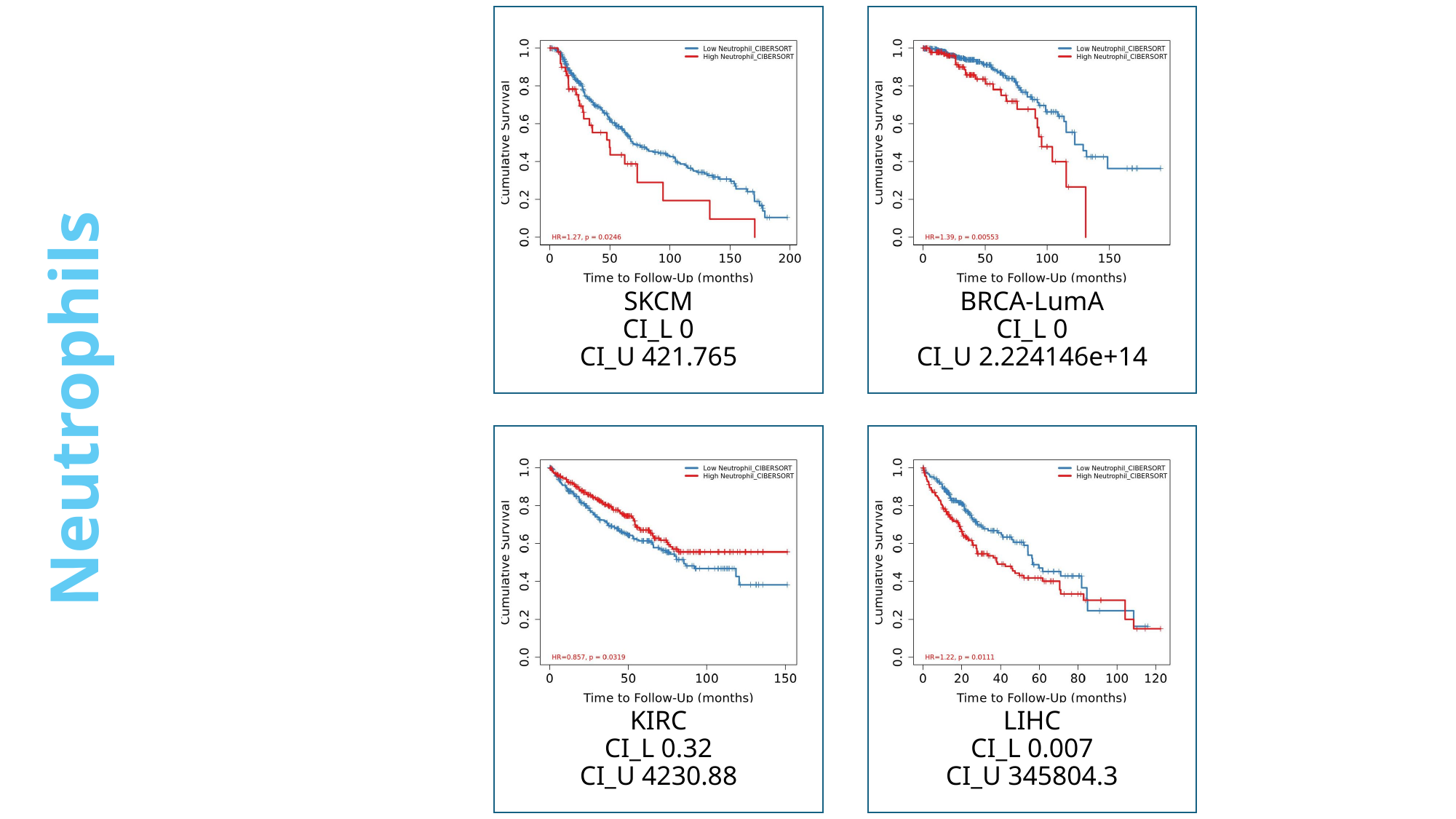

Neutrophils

## Slide 13
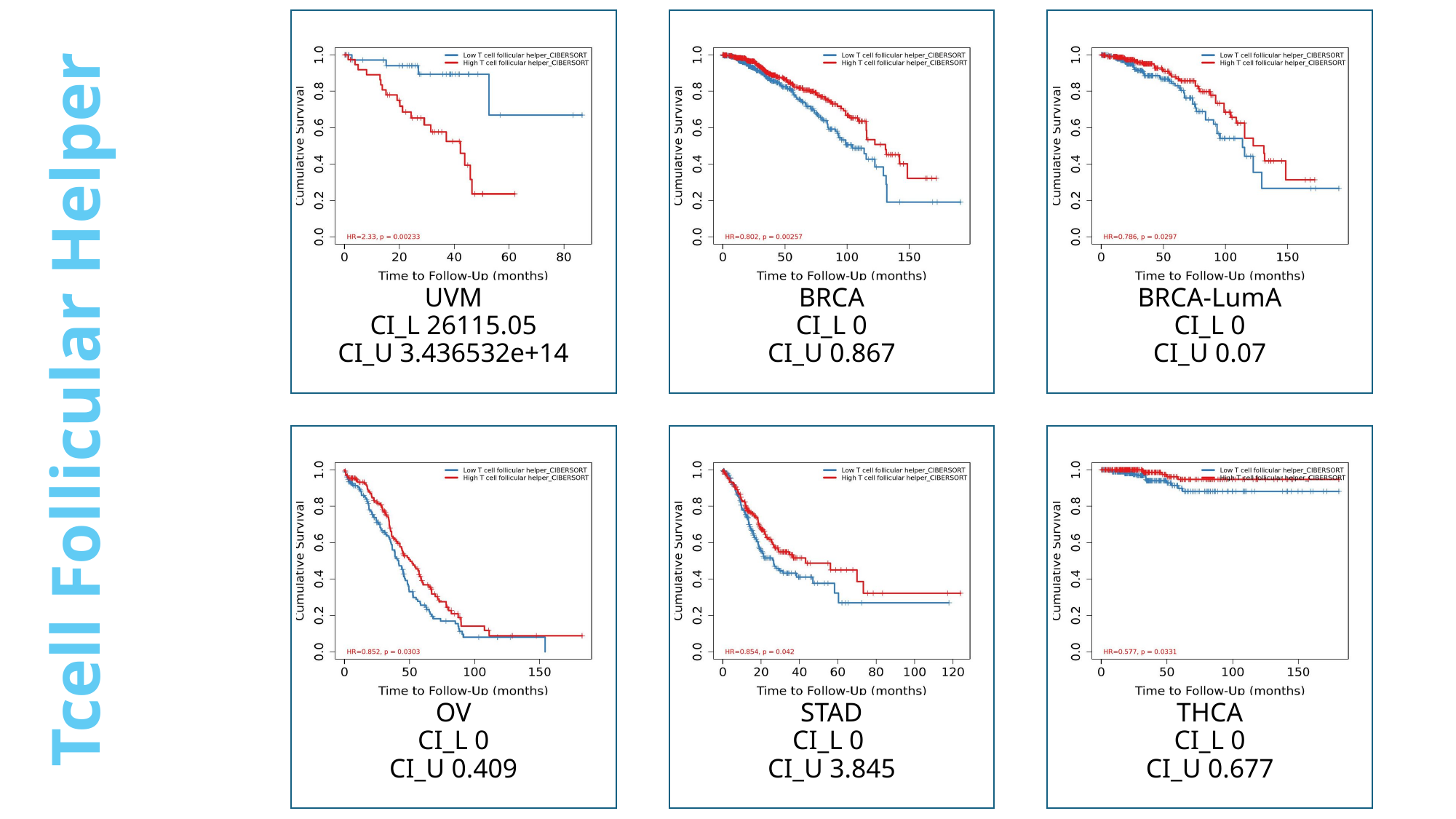

Tcell Follicular Helper

## Slide 14
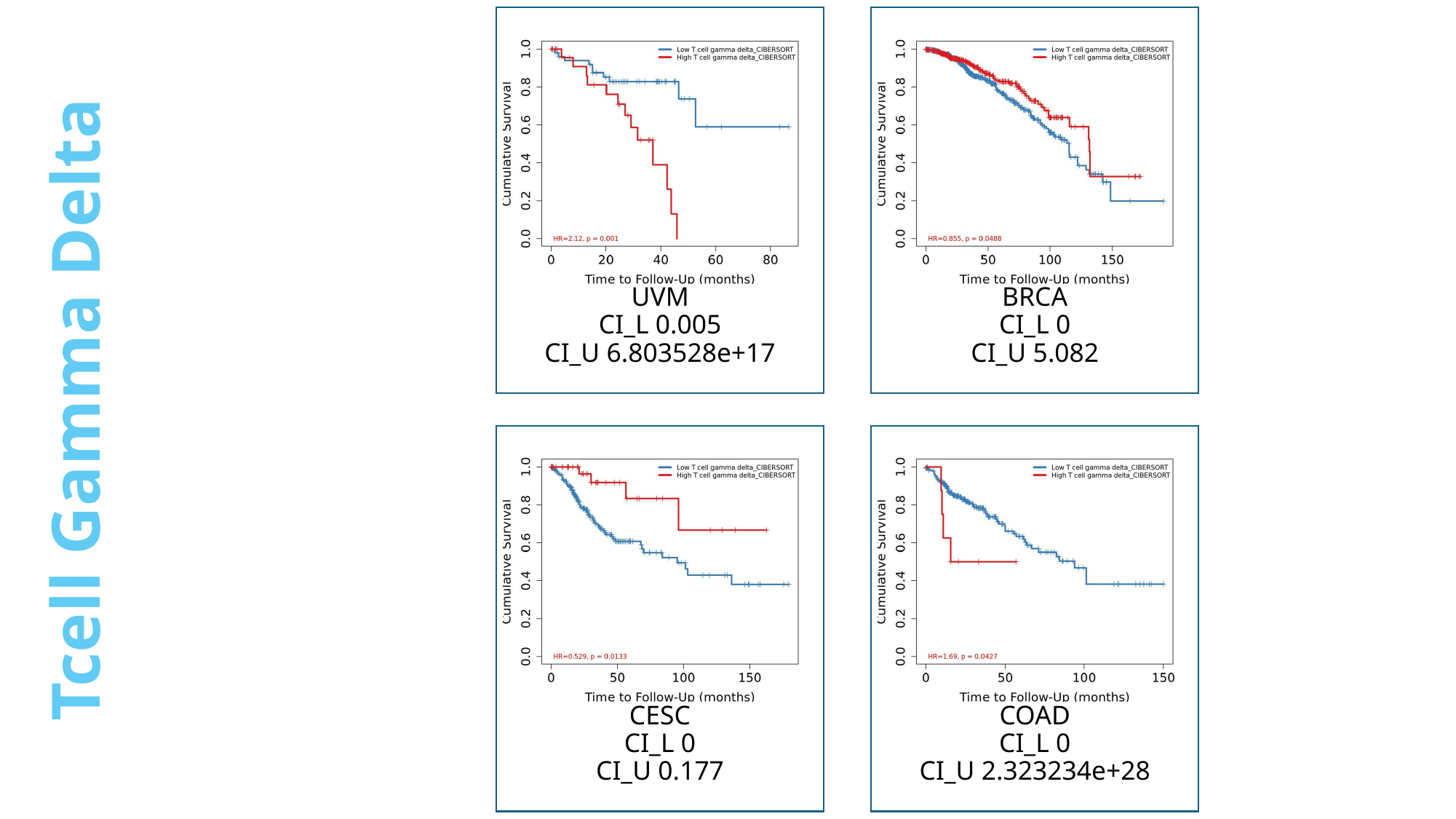

Tcell Gamma Delta

## Slide 15
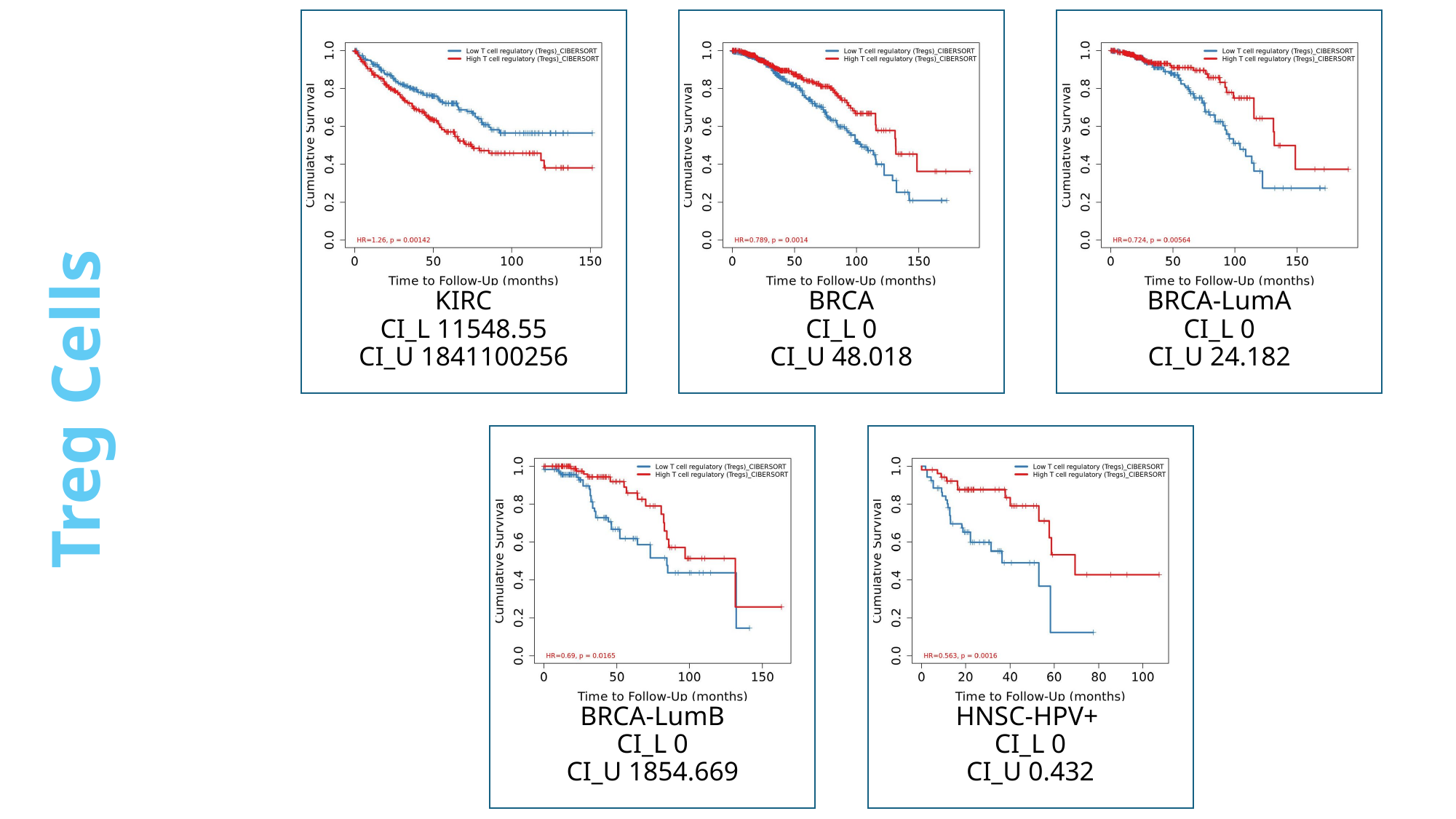

Treg Cells
