# Supplementary material for: Modulatory effect of metformin and its transporters on immune infiltration in tumor microenvironment: a bioinformatic study with experimental validation
Source: Discov Oncol. 2025 May 31;16:973. doi: 10.1007/s12672-025-02766-y (PMC12126455; doi:10.1007/s12672-025-02766-y)
Supplement: Supplementary file 5 — Additional file5 [file 12672_2025_2766_MOESM5_ESM.docx]

**Supplementary file 5**

**Venny Intersection Figures**


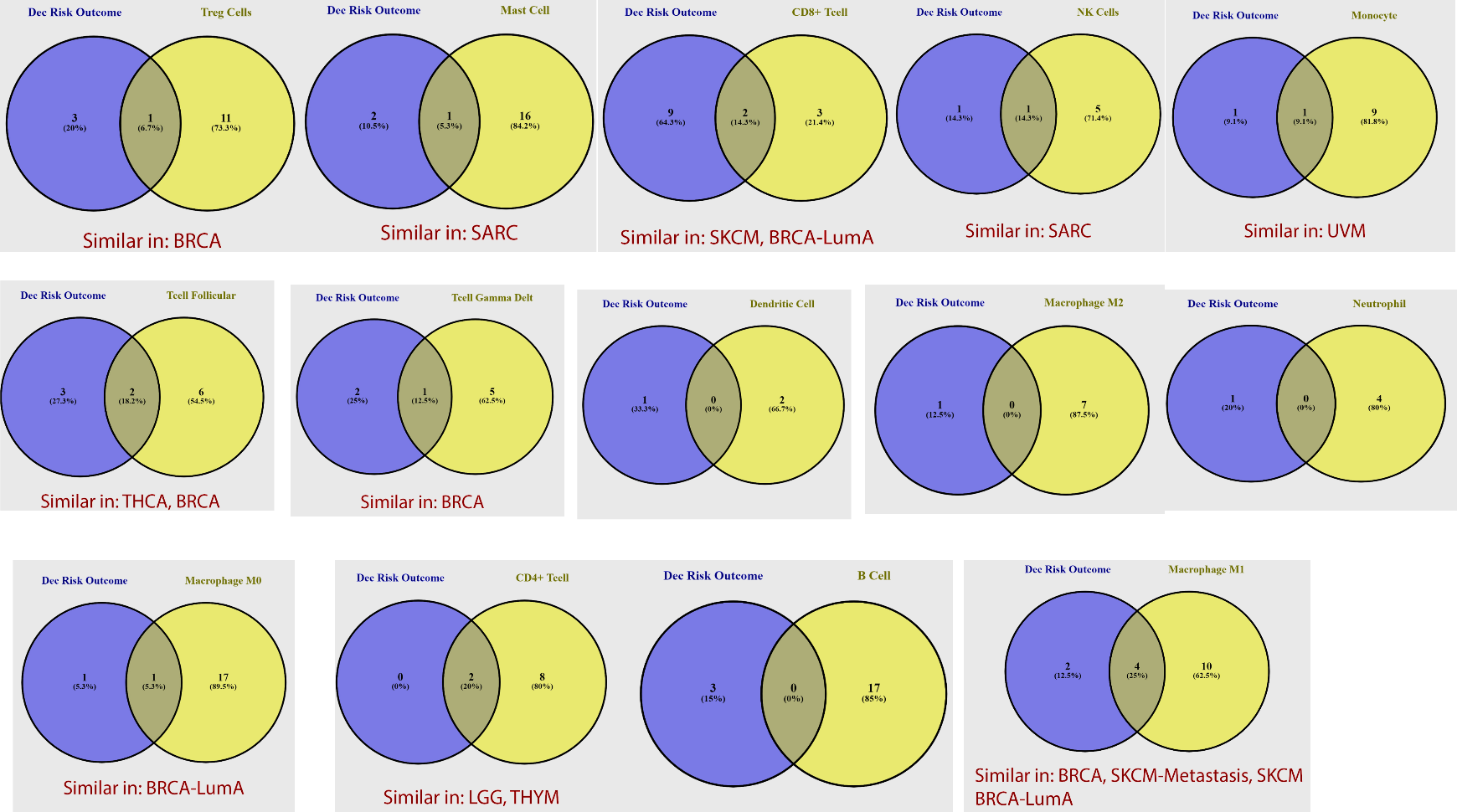


**SI. 1** **Venny intersection results between Immune infiltrates that show decrease risk clinical outcome and different 32 tumor types that showed positive correlation with OCT genes.**
decrease risk immune infiltrates intersected with types of tumors that showed positive correlation with OCT genes.


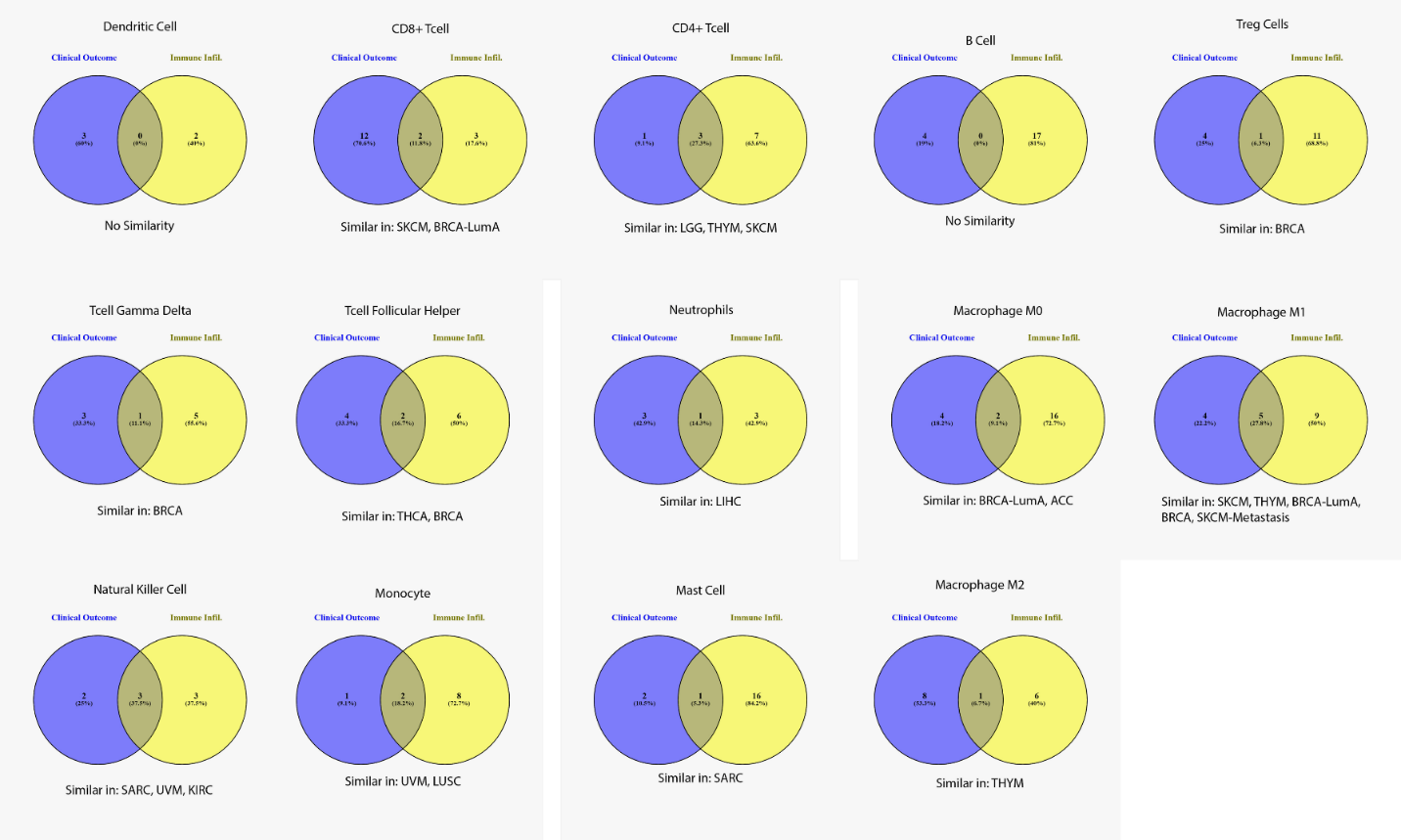


**SI. 2** **Venny intersection results between Immune infiltrates that show both increase and decrease risk clinical outcome and different 32 tumor types that showed positive correlation with OCT genes.**
shows the combined increase and decrease risk immune infiltrates along with the tumor types that had positive correlation with OCT genes.
