# Supplementary material for: Modulatory effect of metformin and its transporters on immune infiltration in tumor microenvironment: a bioinformatic study with experimental validation
Source: Discov Oncol. 2025 May 31;16:973. doi: 10.1007/s12672-025-02766-y (PMC12126455; doi:10.1007/s12672-025-02766-y)
Supplement: Supplementary file 7 — Additional file7 [file 12672_2025_2766_MOESM7_ESM.docx]

**Supplementary file 7**

**OCT genes expression in different TCGA data as compared to normal controls.**The analysis was done using Gene_DE module in TIMER2.0.
Statistical significance computed by the Wilcoxon test that is annotated by the number of stars (*: p-value < 0.05; **: p-value < 0.01; ***: p-value < 0.001).

**OCT 1**


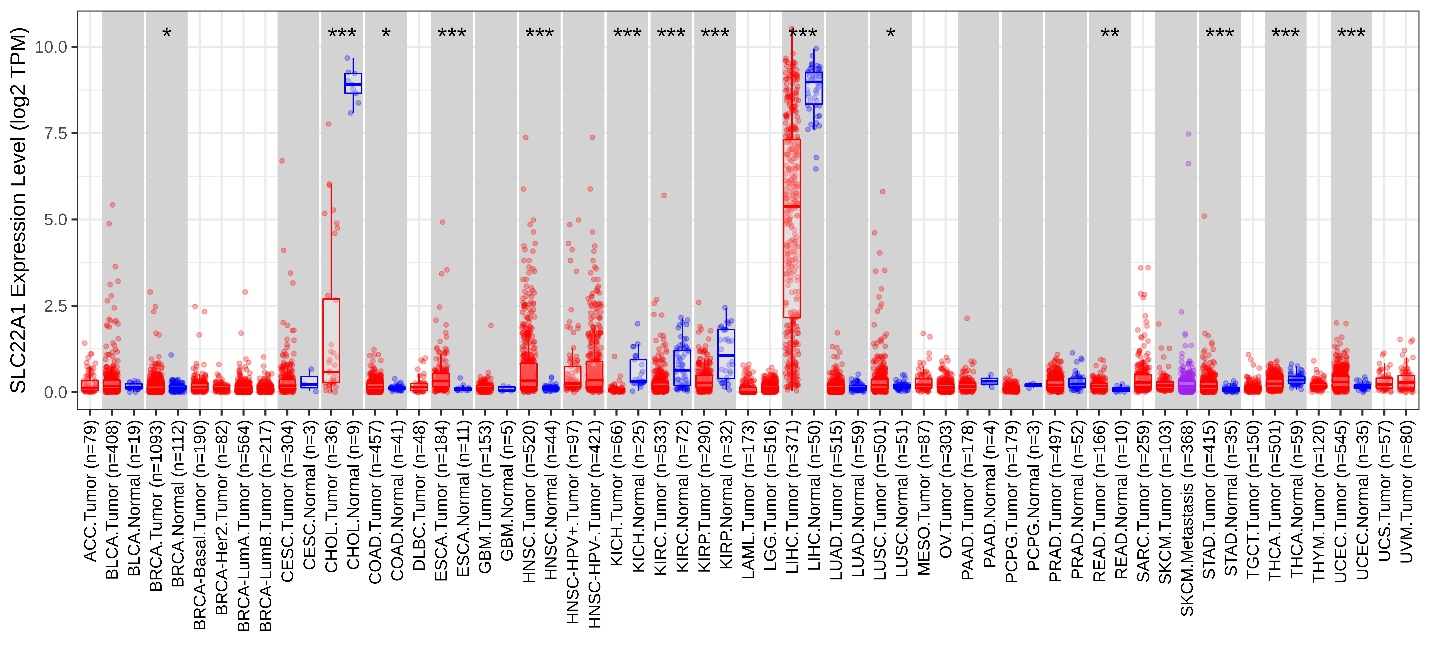


OCT1 = SLC22A1 = Organic Cationic Transporter 1


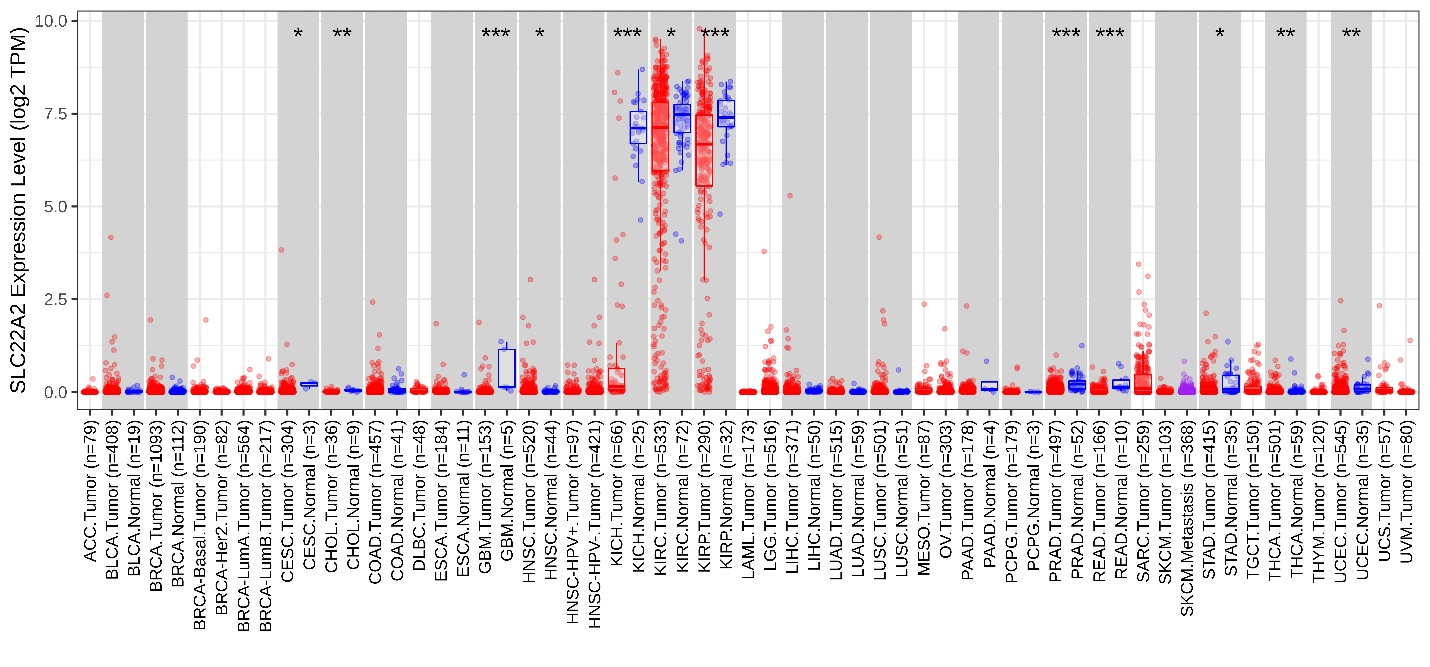


**OCT 2**

OCT2 = SLC22A2 = Organic Cationic Transporter 2


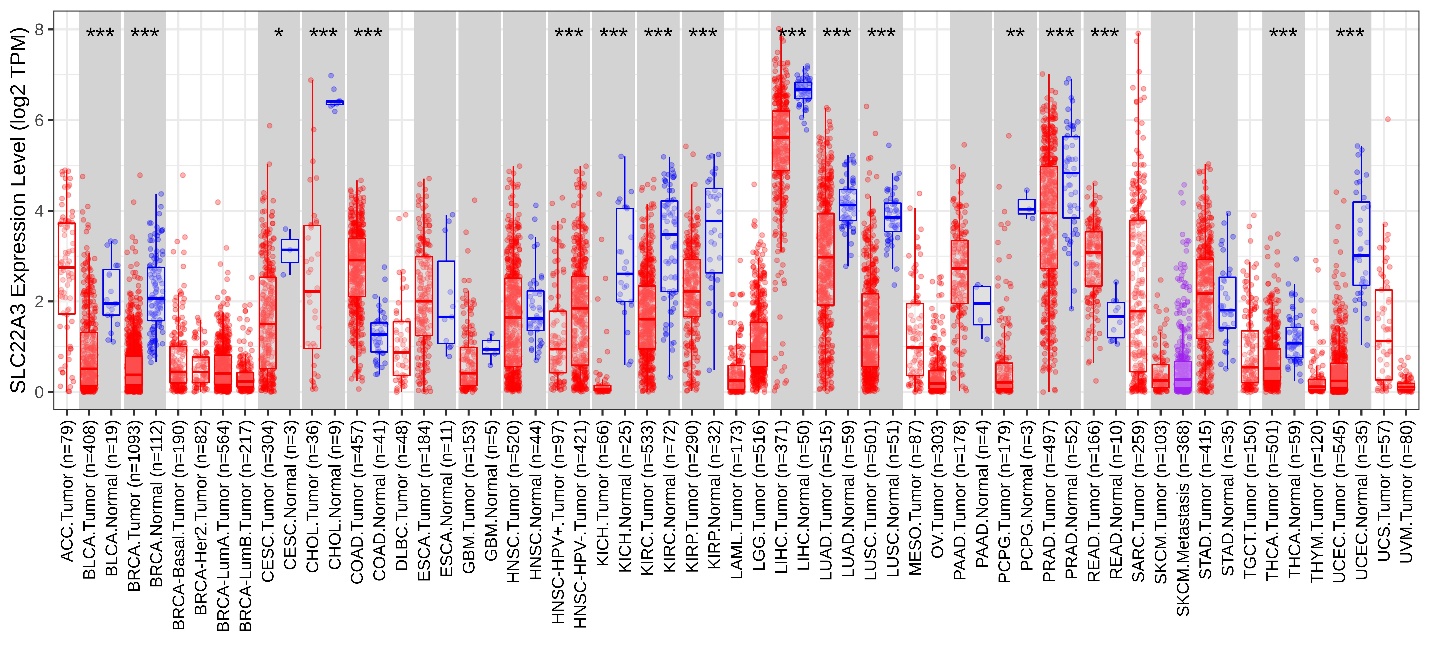


**OCT 3**

OCT3 = SLC22A3 = Organic Cationic Transporter 3
